# Cell State Transition Models Stratify Breast Cancer Cell Phenotypes and Reveal New Therapeutic Targets

**DOI:** 10.3390/cancers16132354

**Published:** 2024-06-27

**Authors:** Oleksii S. Rukhlenko, Hiroaki Imoto, Ayush Tambde, Amy McGillycuddy, Philipp Junk, Anna Tuliakova, Walter Kolch, Boris N. Kholodenko

**Affiliations:** 1 Systems Biology Ireland, School of Medicine, University College Dublin, D04 V1W8 Dublin, Ireland; 2 Stratford College, D06 T9V3 Dublin, Ireland; 3 School of Biological, Health and Sports Sciences, Technological University, D07 H6K8 Dublin, Ireland; 4 Conway Institute of Biomolecular and Biomedical Research, University College Dublin, D04 V1W8 Dublin, Ireland; 5 Department of Pharmacology, Yale University School of Medicine, New Haven, CT 06520, USA

**Keywords:** breast cancer, cSTAR, mathematical modeling, machine learning, signaling networks, network reconstruction, digital twins

## Abstract

**Simple Summary:**

We utilized publicly available perturbation phosphoproteomic data to construct models elucidating cell state transitions across multiple breast cancer and normal breast tissue-derived cell lines. Employing a hybrid methodology, which integrates machine learning and mechanistic modeling, we separated luminal, basal, and normal cell states and revealed core networks that control cell state transitions. We determined causal connections within the core networks and developed interpretable mechanistic models that elucidated the drivers of cell phenotypes. Significantly, these models can predict synergistic drug combinations capable of potentially reversing oncogenic transformation in breast cancer cell lines. Our methodology will enable designer approaches to identify targeted perturbations that convert cell states and mechanistically underpin therapeutic interventions.

**Abstract:**

Understanding signaling patterns of transformation and controlling cell phenotypes is a challenge of current biology. Here we applied a cell State Transition Assessment and Regulation (cSTAR) approach to a perturbation dataset of single cell phosphoproteomic patterns of multiple breast cancer (BC) and normal breast tissue-derived cell lines. Following a separation of luminal, basal, and normal cell states, we identified signaling nodes within core control networks, delineated causal connections, and determined the primary drivers underlying oncogenic transformation and transitions across distinct BC subtypes. Whereas cell lines within the same BC subtype have different mutational and expression profiles, the architecture of the core network was similar for all luminal BC cells, and mTOR was a main oncogenic driver. In contrast, core networks of basal BC were heterogeneous and segregated into roughly four major subclasses with distinct oncogenic and BC subtype drivers. Likewise, normal breast tissue cells were separated into two different subclasses. Based on the data and quantified network topologies, we derived mechanistic cSTAR models that serve as digital cell twins and allow the deliberate control of cell movements within a Waddington landscape across different cell states. These cSTAR models suggested strategies of normalizing phosphorylation networks of BC cell lines using small molecule inhibitors.

## 1. Introduction

In the past, biochemistry and biophysics “decomposed” a living cell to focus on single proteins, enzymes, ion channels, and receptors, whereas classic physiology explored the responses of an entire organ or organism to stimuli, treating the objects under study as systems. The rise of omics technologies has revived the systems view of classic physiology by emphasizing the fundamental challenge of understanding how a vast compendium of gene and protein expressions and activities is integrated in cellular networks that generate cell fate decisions and phenotypes. A key challenge of 21st century biology is to predict a cell’s phenotypic behavior from molecular profiles that can be generated by the ever more prolific omics technologies. However, at present, the precise prediction of drug responses, cell fate decisions, and transitions between cell states, based on cell molecular profiles is elusive. Although omics data acquisition advances at a formidable pace, our ability to mechanistically interpret omics datasets and elucidate how they dictate cellular phenotypes is still trailing behind.

Recent advances in single cell RNA sequencing (scRNAseq) led to the development of elegant machine learning (ML) and dynamics-based approaches to predict cell trajectories in the RNA space and extract information about gene interactions [1,2,3,4]. Yet, simple genotype–phenotype associations are often flawed because they neglect the impact of the complex regulatory networks that connect the genotype with the phenotype. External and internal cues processed by cell-surface receptors and downstream signaling pathways and resulting changes in posttranslational protein modifications, such as protein (de)phosphorylation, connect a cell’s biological responses and state transition dynamics with a cell’s omics profiles. The current challenge of systems biology, network analysis, and computational approaches is to predict the perturbation outcomes and cell fate decisions by extracting dynamical and mechanistic information from a limited number of (phospho)proteomic and transcriptomic snapshots.

Here we explore a strategy to study and deliberately control the cellular phenotype using a cell State Transition Assessment and Regulation (cSTAR) approach [5]. We utilized a mass cytometry (CYTOF) dataset, which describes single cell phosphoproteomic patterns of 62 breast cancer (BC) and five normal breast tissue cell lines under five different kinase inhibitor perturbations [6]. Based on these data, cSTAR unambiguously separates the phosphoproteomic states of BC cell lines and the cell lines developed from normal breast tissue, as well as luminal BC cells vs basal BC cells. The segregation of a huge variety of cell states is demonstrated to be robust (see Section 3.3 and Section 3.4 in Results). Following cell state separation, we delineated core signaling networks that control phosphoproteomic and phenotypic states across both BC and normal cell lines. We then reconstructed causal network connections, as accurately as the available CYTOF data allowed. Finally, using cSTAR we built mechanistic models describing signaling and phenotypic responses to drug perturbations. It has enabled us to establish ways of controlling cell phenotype, such as switching BC cells between different subtypes and reversing or promoting oncogenic transformation. 

The analysis of cSTAR data-driven models reveals intriguing molecular features of BC cells. Although 33 different luminal BC cell lines have distinct mutational and expression profiles, the inferred architecture of causal connections of core networks in these cell lines is similar, and oncogenesis is mainly driven by the mTOR pathway. In sharp contrast, the inferred connection circuitries of core networks among 28 distinct basal BC cells exhibit significant qualitative differences, partitioning into mainly four different classes with different driver pathways. Surprisingly, the inferred connection circuitries within core networks of non-malignant breast epithelial cells are also separated into two qualitatively distinct classes. Our cSTAR models have allowed digital cell twins to be created that describe how oncogenic states are maintained in each BC cell class, analyze cell maneuvering in Waddington’s landscape [7], and suggest experimental strategies of normalizing the cell’s phosphoproteome. We discuss how these new strategies are supported by the available data. We describe each cSTAR step in detail to demonstrate that it is a new generalizable framework, which enables biologists to unravel the cell fate decision machinery using systematic perturbations, precisely predict cellular network responses, state transitions, and ultimately deliberately control cell phenotypes. We also show that cSTAR can be applied to biological problems that feature multiple cell states.

## 2. Materials and Methods

### 2.1. Pre-Processing the CYTOF Data

We used the raw CYTOF data in the fcs-format available at Mendeley Data (https://doi.org/10.17632/gvh2vtg86r.1, accessed on 26 June 2024) [8]. The uploaded fcs files had outputs of 29 antibodies. The data were sorted into multiple batches [8]. Each batch included (i) a full set of perturbation data for one or two cell lines under Epidermal Growth Factor (EGF) stimulation of serum-starved cells, (ii) data from the same cells growing in serum, and (iii) the data for HCC70 cell line measured under serum-starved conditions For each sample, each antibody and every batch, we calculated the average intensity value, and normalized it to the average signal level for this antibody in the batch control, i.e., HCC70 cell line data within the same batch.

All CYTOF data after normalization and pre-processing are given in Supplementary Table S1.

### 2.2. Building the STVs and Calculating the DPD Scores

We applied the SVM algorithm with a linear kernel to the data from cells growing in serum to separate (1) BC cell lines and non-malignant breast epithelium tissue-derived cell lines and (2) BC cell lines of luminal BC subtype and BC cell lines of basal BC subtype. Cell line labeling, both normal vs cancer, and luminal vs basal breast cancer, were taken from the original publication [8]. The confusion matrix was diagonal in both cases, indicating flawless separation of cell states. After we built the two separating, maximal margin hyperplanes, we constructed the two unit vectors that are normal to these two separating hyperplanes. We called these vectors the State Transition Vectors, the STV_onc_ and the STV_LB_ (often, the STVs can also be derived using the vectors that connect the centroids of clouds of data points [5]). After building the STVs, cSTAR calculates the Dynamic Phenotype Descriptor (the DPD_onc_ and the DPD_LB_) scores, which describe the changes in the cell states that would occur when the data point crosses the separating hyperplanes. The absolute value of each DPD score is defined as the signed distance function from a cell data point to the separating hyperplane. The positive or negative sign of the DPD score indicates if the direction from the data point to the hyperplane is parallel or antiparallel to the corresponding STV [5]. Formally, the separating hyperplane built by SVM is defined as follows:

(1) $$x\cdot n_{s}=h_{s},$$

The corresponding DPD score, ${DPD}_{s}^{i}$, is calculated for each data point $x_{i}$ using the following expression:

(2) $${DPD}_{s}^{i}=-\left(h_{s}-x_{i}\cdot n_{s}\right)$$

Here $n_{s}$ is the corresponding STV, and index “s” corresponds to either DPD_onc_, or to DPD_LB_. The vector $n_{s}$ and the scalar $h_{s}$ are supplied by the SVM algorithm. Supplementary Tables S2 and S3 contain components of the STV vectors and the calculated DPD scores.

In line with the cSTAR approach [5], after determining components of a core network (see main text), we removed the proteins related to the core network modules from the dataset and re-calculated the STV vectors and the DPD scores. To improve the classification of cell states, we left pS6 and pSTAT3 both in the DPD module and in the core network due to the low number of measured analytes (low dimensionality of the data of only 29 antibodies). To maintain the modular insulation condition [9], the Bayesian Modular Response Analysis (BMRA) perturbation matrix [5] denotes that mTOR inhibitors perturb both the mTOR and DPD modules. Nevertheless, a minor amount of non-zero, non-diagonal elements in the confusion matrix was present for DPD_LB_, indicating slight inaccuracy in the classification of BC subtype cell states, brought about by the low data dimensionality.

### 2.3. Reconstruction of Core Networks

A complete description of the BMRA network reconstruction algorithm is given in Ref. [5]. Briefly, BMRA requires the following matrices as an input: the global response matrix $R_{ij}$, a prior for network topology and the perturbation matrix, which specifies the nodes that were directly perturbed for each sample. The perturbation matrix was calculated based on the description of perturbation experiments in ref. [6].

For the core network components, the elements of the global response matrix $R_{ij}$ were calculated as follows [10]:

(3) $$R_{ij}=2\frac{x_{1}-x_{0}}{x_{1}+x_{0}}$$

Here $x_{0}$ and $x_{1}$ are the module outputs for the same time point (after EGF stimulation) with no and with drug perturbation, respectively. The list of module outputs is presented in Supplementary Table S4.

For the DPD modules, elements of the global response matrix were calculated as below:

(4) $${\;\;\;\;\;R}_{ij}=\frac{{DPD}_{s}^{1}-{DPD}_{s}^{0}}{\left|{DPD}_{s}^{0}\right|}$$

Here the ${DPD}_{s}^{0}$ and ${DPD}_{s}^{1}$ are the DPD values for the same time point following EGF stimulation without and with drug perturbation, respectively.

For building consensus networks, we constructed the global response matrix $R_{ij}$ by concatenating the global response matrices corresponding to different cell lines. We slightly modified the BMRA algorithm by allowing both to add to and delete connections from a prior network. To prevent prohibited connections, as delineated in cSTAR, from the DPD modules to signaling modules [5], we incorporated the table of forbidden connections in the input to BMRA. We constructed the prior network topology matrix assuming the absence of pathway cross-talk and allowing BMRA to identify the most important cross-talk connections. The prior network topology matrix is presented in Supplementary Table S5. The python code preparing the BMRA input is available in the supplementary archive “BC_CYTOF_preprocessing.zip”. We used a 20% Occam’s window in BMRA sampling. Matlab code implementing BMRA is provided in the supplementary archive “BMRA.zip”. BMRA-reconstructed local response matrices were used for further analysis and model development.

### 2.4. Model Construction and Simulation

We used the BMRA-reconstructed networks to build nonlinear dynamical models for different cell lines. The models were constructed using the PySB framework [11]. In these models, core network modules/proteins can be in active and inactive states, and cross-talks between modules are generally described by the hyperbolic multipliers [12]. We consider interactions exclusively between those modules where the BMRA-inferred connection coefficients demonstrate statistically significant non-zero values. 

To recapitulate better the existing experimental data on the activation of the MAPK/ERK pathway, a connection from EGFR to ERK was modelled in greater mechanistic detail. We included the following biochemical events into a description of the EGFR to ERK pathway (see Supplementary Figure S1: (1) EGF-EGFR interaction, (2) EGFR dimerization and autophosphorylation, (3) SOS recruitment to the plasma membrane by active EGFR, (4) RAS-GDP to RAS-GTP exchange by SOS, (5) RAF activation by RAS-GTP, (6) MEK phosphorylation by active RAF, (7) ERK phosphorylation by active MEK, (8) ERK phosphorylation on SOS and RAF that suppress activities of these proteins.

To replicate the experimental setup of EGF stimulation following serum-starvation, the model was initially simulated without EGF until reaching equilibrium. We took the final state of the initial equilibrium simulation and used it as the initial condition of new simulations with growth factors and kinase inhibitors. These simulations were used for a fitting procedure to determine model parameters. Specifically, time-series data for pMKK4, pAKT, pGSK3B, pRSK, pS6, pSTAT3, and the DPD values were collected under EGF treatment conditions, both with and without kinase inhibitors. Models were simulated using the ScipyOdeSimulator provided by the PySB framework. The experimental data were fitted using a sum of squared residuals objective function. Because the optimization problems can contain multiple local optima, to minimize efficiently the objective function, we used the differential evolution method [13] provided by SciPy [14]. The matplotlib [15] and seaborn [16] packages were used for the visualization of the results.

### 2.5. Parameter Estimation of Cell Line-Specific Models

We utilized the time courses of the phosphorylation levels of MKK4, AKT, GSK3β, RSK, STAT3, RPS6, and the two DPD modules (DPD_onc_ and DPD_L-B_) post-EGF stimulation to estimate the model parameters (Supplementary Figures S2–S4). For each cell line-specific model, this was achieved by minimizing the sum of squares of the differences between the experimental and simulated data points using the Differential Evolution algorithm [13] provided by SciPy [14]. Five optimization runs were performed in parallel using randomly sampled initial parameter values. We selected the parameter set with the smallest objective function value for the simulations. The resulting model parameters are available in the SBML files in the Supplementary Materials.

### 2.6. Construction of Waddington’s Landscape

Our cSTAR models include the DPD modules that describe how the upstream signaling modules affect cell states [5]. The DPD modules are governed by two different types of forces: the signaling force and the gradient restoring force. The signaling force σ(t) is determined by the activation dynamics of the signaling modules that directly affect the DPD modules:

(5) $$\sigma \left(t\right)=\sum_{j}r_{Sj}\left(\frac{S_{st.st}}{x_{j}^{st.st}}\right)x_{j}\left(t\right)$$

Here, *S* is the DPD_onc_ or DPD_L-B_, ${\;x}_{j}(t)$ are the activation dynamics of signalling modules, $r_{Sj}$ are the corresponding BMRA-inferred connection coefficients to the DPD_onc_ or DPD_L-B_, and $S_{st.st}$ and $x_{j}^{st.st}$ are the initial steady state values of S and $x_{j}$ before perturbations.

The restoring force f(S) is the gradient force given by the derivative of the potential (U):

(6) $$f\left(S\right)=-\frac{dU}{dS}$$

We assume that each phenotype corresponds to a stable steady state and apply the parabolic potential (U), which is widely used in the field of physics [17]. The potential U has two minimums, which correspond to the two steady states that are characterized by the DPD scores, $S_{0}$ and $S_{1}$. These steady states are stable; therefore, after small disturbances, the system returns to the original stable state. The scores $S_{0}$ and $S_{1}$ take negative and positive values, respectively. There is an unstable steady state at the boundary between the $S_{0}$ and $S_{1}$ attractor regions. 

Accordingly, the restoring force is modelled using a piece-wise linear approximation:

(7) $$f\left(S\right)=\left\{\begin{array}{cc} -\alpha_{0}(S-S_{0}), & \;\;S<\frac{3S_{0}+S_{1}}{4}\\ \alpha_{0}(S-\frac{S_{0}+S_{1}}{2}), & \;\;\frac{3S_{0}+S_{1}}{4}<S<\frac{2S_{0}+{2S}_{1}}{4}\\ \alpha_{1}(S-\frac{S_{0}+S_{1}}{2}), & \;\;\frac{2S_{0}+{2S}_{1}}{4}<S<\frac{S_{0}+{3S}_{1}}{4}\;\\ -\alpha_{1}(S-S_{1}), & \;\;\frac{S_{0}+{3S}_{1}}{4}<S \end{array}\right.$$

Here, the stable steady state positions $S_{0}$ and $S_{1}$ correspond to (i) normal and oncogenic, or (ii) luminal and basal states for the DPD_onc_ and DPD_L-B_ scores, respectively. The DPD trajectory is obtained from the following equation:

(8) $$\;\frac{dS}{dt}=f\left(S\right)+\sigma \left(t\right)=-\frac{dU}{dS}+\sum_{j}r_{Sj}\left(\frac{S_{st.st}}{x_{j}^{st.st}}\right)x_{j}\left(t\right)$$

This gradient force field and the field of external forces shape the evolving Waddington’s landscape (W), as follows:

(9) $$W=U-\sum_{j}r_{Sj}\left(\frac{S_{st.st}}{x_{j}^{st.st}}\right)x_{j}\left(t\right)\cdot S\left(t\right)$$

The slope parameters ($\alpha_{0}$ and $\alpha_{1}$), stable steady state positions ($S_{0}$ and $S_{1}$), and the coefficients $\beta_{j}=r_{Sj}\left(\frac{S_{st.st}}{x_{j}^{st.st}}\right)$ are estimated from phosphoproteomic time-course data during model parameterization.

## 3. Results

### 3.1. Combining Machine Learning, Network Reconstruction and Mechanistic Modeling to Understand and Manipulate Cell States—The cSTAR Approach in Action

A comprehensive catalogue of somatic mutations and copy number aberrations involved in BC pathogenesis is currently available [18,19,20]. However, how these mutations affect biochemical network functions in the cancer cells, and specify disease manifestation is unknown [21,22]. Cellular identity arises from a myriad of combinatorial interactions between molecules in the cell. The concentrations or activities of these molecules, termed analytes, determine cell states and can be measured by different omics techniques. Here we show that the cSTAR approach allows us to create digital cell twins of cells that accurately describe and distinguish complex cellular phenotypes and help us to deliberately control cell fate decisions.

### 3.2. Applying Supervised ML to Separate Distinct States of BC and Non-Malignant Breast Cells

The first step in the cSTAR pipeline is to distinguish different cell states employing ML-based classifiers. Unsupervised learning is often applied to reveal cell states using omics data. Various methods are frequently used, including principal and independent component analyses (PCA and ICA, respectively), hierarchical clustering, the minimum spanning tree, Monocle, and other related techniques [23,24,25,26]. These methods assume the equal importance (weight) of each measured analyte, and the directions of the highest data variation often reveal main features underpinning different cell states. These unbiased assumptions are logical and justified, if there is no a priori knowledge about cell features. Yet, additional information often exists. For instance, morphological features are widely used to characterize cell states and phenotypes under different conditions and to identify transformed cells [27,28]. Also, mutations that are known oncogenic drivers may change weights or scores that describe the importance of analytes. By applying supervised algorithms, such as K-Nearest Neighbors (KNN), Support Vector Machines (SVM) with different kernels or Random Forests (RF), we can accurately estimate the weight of each analyte and design nearly flawless cell state classifiers. In RF, the analyte weight is built into the algorithm [29], whereas in the SVM and KNN it can be determined by temporarily removing each analyte and estimating the precision of classification [30]. The SVM with a linear kernel is the best performer when the data dimensionality greatly exceeds the number of observations [31], as it is always the case for omics data. When the data dimensionality is comparable with the number of observations, then KNN or SVM with different non-linear kernels show the most accurate results.

**Figure 1:**
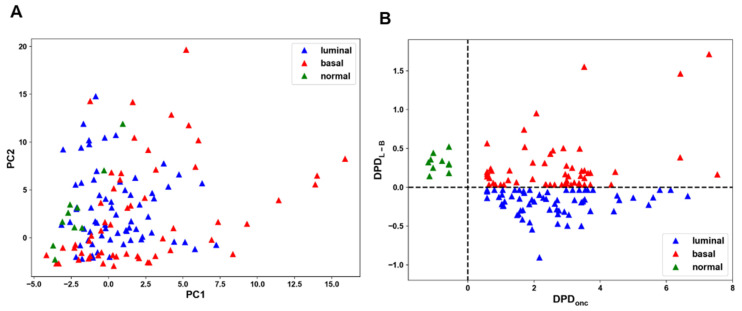
A failure of PCA to separate cell states and their robust separation by cSTAR. (**A**) PCA fails to separate different cell states for CYTOF dataset of 61 BC and 5 non-malignant breast epithelial cell lines growing in serum. (**B**) cSTAR separation of cell states for different BC types (red and blue triangles) and normal breast epithelial-derived lines (green triangles) in the DPD_onc_ and DPD_L-B_ space. The DPD scores equal to zero correspond to the separating hyperplanes (shown by dashed lines).

To test the performance of these classifiers to distinguish different cell states we reanalyzed a single-cell phosphoproteomic dataset of serum and EGF stimulated 61 BC and five normal tissue-derived lines (following data normalization, 61 from 62 BC lines were left) [6]. Distinct cell states were hardly separable [6]. Hierarchical clustering could not distinguish BC from non-cancer and luminal from basal cell subtypes, using the median values (see Supplementary Figure S2 in reference [6]) or average values (Supplementary Figure S5) of mass cytometry intensities of each antibody. Likewise, PCA is unable to separate the signaling patterns of BC cell lines growing in serum (Figure 1A). Of note, only the total protein expression profiling proteomic data allowed PCA separation of luminal vs. basal BC cells [6]. However, this separation is limited to changes in protein abundance and does not allow us to understand signaling processes controlled by phosphorylation, which play major roles in cell fate decisions [32].

To separate robustly (i) BC cell lines and lines developed from normal tissue, and (ii) luminal vs basal BC subtypes, we trained the SVMs with linear kernels, using the average values of mass cytometry intensities of phosphosites detected in these cell lines growing in serum and subsequently, the SVMs built separating, maximal margin hyperplanes (Methods). The data confusion matrices were diagonal, showing perfect separation, whereas a cross-validation demonstrated its high robustness, as described below.

### 3.3. Testing the Robustness of the cSTAR Predictions

We assessed the robustness of our cSTAR predictions based on a constrained set of 29 phospho-protein markers, using the following analyses.

First, we applied an 8-fold cross-validation (CV) [33] for the SVM classification of cell states after removing data related to one cell line. The precision of classification of cancer vs non-cancer cells was 96%, and the precision of the classification of the BC subtype was 70%. The python code performing all these operations is available in the supplementary archive “BC_CYTOF_preprocessing.zip”.

Second, we introduced in silico perturbations to the dataset by removing individual analytes for the classification of cell subtypes. The effect of analyte removal was evaluated using an 8-fold CV. The results showed that the accuracy did not change for both cancer vs. non-cancer and luminal vs. basal classifications after eliminating an analyte (Supplementary Figure S6). This indicates that the cSTAR predictions remain consistent even after the removal of any analyte from the CYTOF dataset.

Third, we investigated the robustness of cSTAR predictions by adding artificial noise to the dataset. The magnitude of noise was a certain percentage ($n_{r}$) of the measured value for each analyte, so that the signal to noise ratio was equal to $1/n_{r}$. The value (x) of each measured analyte was modified by adding a uniformly distributed random value within a range between $-n_{r}\cdot x$ and $n_{r}\cdot x$ where $n_{r}$ is a positive number between 0 and 1. For each $n_{r}$, we ran an 8-fold CV iteratively for 1000 repetitions. We noted that even in the extreme case of highly noisy data with the added 50% noise ($n_{r}=0.5$), there was no discernible change to the mean accuracy and the interquartile did not exceed 15% of the median (Supplementary Figure S7). 

In summary, the cSTAR analysis demonstrated high robustness in analyzing oncogenic transformation, specifically the transition from non-malignant to malignant cell states. However, there was diminished confidence in predicting BC subtypes, particularly the transitions between luminal and basal-like subtypes. 

### 3.4. The Accuracy of BC Cell State Classification Using Total Proteomics Data

One of the prerequisites of cSTAR is to have an ample quantity of measured proteins or genes for cell state classifications and for constructing the STVs. Given the limited number of measured analytes in CYTOF data [6], totaling 29, we investigated the impact of the number of analytes on classification accuracy using mass spectrometry proteomic data. We randomly selected $n_{markers}$ (10, 20, 30, 100, or 300) proteins from a dataset containing 7995 measured proteins. Next, we employed an 8-fold cross-validation to compute the mean accuracy. This process of sampling and cross-validation was iterated 1000 times. We could discern between non-cancerous and cancerous cells with fewer than 100 analytes. However, a greater number of analytes was necessary to distinguish between luminal and basal breast cancer cell lines, as depicted in Supplementary Figure S8A. We observed a large variance in the accuracy when $n_{markers}=10$, and found that the accuracy depends on which analytes to select. When we randomly sampled analytes, constructed the STV, and calculated the DPD scores, certain cells were classified incorrectly (Supplementary Figure S8B). On the other hand, the ten proteins constituting the core network accurately classify cells (Supplementary Figure S8C). Overall, both the quantity and specificity of the analytes are pivotal factors in accurately classifying cells. Given our primary objective of distinguishing between normal and cancer cell states, the constrained number of analytes measured in the CYTOF dataset [6] and used by cSTAR was sufficient to provide ample insights into cell transitions between oncogenic and normal states. 

### 3.5. Building the State Transition Vector (STV)

The SVM cell state separation implies a geometrical interpretation where the maximal margin hyperplane separates clouds of data points corresponding to different cell states and phenotypes [34]. In this multidimensional molecular space, the direction of a transition from one cell phenotype to another can be determined by a vector that connects the centroids of these two data point clouds or by the shortest path along the vector normal to the separating hyperplane. These two directions are closely correlated, and either one can be selected as a direction of cell state transition [5,35]. As we separated cancer vs non-cancer cells and luminal BC vs basal BC, we obtained two cell state separating hyperplanes. Accordingly, we built two normal vectors, (i) from BC states to cell states from normal breast tissues and (ii) from luminal BC to basal BC. These vectors built in the phosphoproteomic data space are termed the STV_onc_ and STV_L-B_, respectively [5,35]. The absolute values of the STV components quantify the importance of the changes in each molecular feature space to the overall change in molecular data that leads to a transition between separated cell states [36].

### 3.6. Determining Components of a Core Controlling Network

Our key hypothesis is that multiple molecular changes accompanying cell state transitions and captured by the DPD changes are determined by a handful of key components, which compose a core controlling network. This hypothesis is partly based on the “enslavement principle” known in physics [37], stating that the behavior of a complex system in the vicinity of the change of its state is guided by a small number of parameters.

The STV components capture the contributions of individual molecular features to the direction of cell state change. We take the absolute values of the contributions to each of the two STVs: (i) the STV_onc_ that determines the direction of oncogenic breast tissue transformation, and (ii) the STV_L-B_ that specifies the direction from luminal BC type to basal BC type (Supplementary Table S2). The highly ranked components constitute the core network that controls the changes in the cell-wide network and the phenotypes scored by the DPDs. Importantly, each node of a core network can be a single protein or entire pathway whose activation output is described by the activity of this protein [10,38]. To cut off a list of the STV components, which constitute the core network, prior knowledge and bioinformatic enrichment analyses are useful. Including more STV constituents increases the granularity, but it also increases the number of perturbations needed for the subsequent reconstruction of causal network connections. Hence, the cut-off for components to include in the core network depends on the available perturbation dataset and the desired granularity. Such a semi-manual cut-off and selection strategy is used in many multiscale methods [39]. The components of the core control network must be perturbed to infer causal connections that shape the network dynamics and output.

Our core network combines high-ranked components of both STV_onc_ and STV_L-B_ for luminal and basal BC cell lines and normal breast tissue-derived lines. Because only five kinase inhibitor perturbations were measured [6], we confined our core network to include the following six biochemical modules/pathways, PKCα/PKCβ/GSK3β, MEK/ERK, PI3K/AKT, EGFR, mTOR/S6K, and STAT3. This is a reasonable compromise given the available CYTOF dataset with only 29 measured phosphosites and five inhibitor treatments. The remaining components of the cell-wide network comprise the two DPD nodes that specify two different cell phenotypic features [35]. The reduction in the number of analytes utilized to compute the DPD scores did not impact the precision of detecting oncogenic transformation, yet it decreased precision in the detection of BC subtypes, as outlined in the Section 3.3.

Whereas core network molecular components are described by their concentrations or activity levels, the DPD nodes are described by the DPD scores. The changes in the oncogenic transformation score, DPD_onc_, occur along a path between non-transformed and BC states, and the changes in the DPD_L-B_ score relate to the change in a cell state along a path between luminal and basal cancer types, respectively (Figure 1B). Because the DPDs connect molecular features to the phenotypes, the logical next step is to predict how to control and purposefully manipulate the DPD scores, thereby engineering the cell phenotype.

### 3.7. Mechanistic Insights Coming from Core Network Reconstruction Explain Predictions and DPD Scores

Multiple reports link different activation patterns of the same proteins, including ERK, JNK, AKT, p53, to distinct cell fate decisions and phenotypic outcomes [40,41]. The different activation patterns of the core network components, which control a cell-wide network, are determined by the quantitative topology (in other words, circuitry) of the core network [42,43]. This topology is defined by causal connections and their strengths [44]. Recent data show that the connections can be different for cells with almost identical genetic background, and their strengths can change with changes in environmental cues, stimulation time, and cell states [42,45]. Accordingly, we examined the core networks and the causal connections in distinct cell states at different time points. 

### 3.8. Modular Response Analysis of Cell Function

We previously developed a physics-based method, Modular Response Analysis (MRA), to reconstruct exactly and quantify causal connections between network nodes, including feedback loop connections, using systemic perturbations [10,46,47]. A connection from one network module (j) to another (k) is quantified as the fractional change in the output of the affected module k brought about by a 1% change in the output of the effector module j when both these modules are considered in isolation from the network. Therefore, in the MRA framework, any causal connection has a direction and strength quantified by the connection coefficient, and the network topology is quantified by the matrix of connection coefficients (also known as local responses) [48]. The connection coefficient can also be referred to as connection strength. The connection coefficients cannot be directly measured, because a perturbation to any module rapidly propagates, blurring the causality of connections [38,49]. Bioinformatic inference algorithms, such as those based on random forests (GENIE3) and mutual information (ARACNE), can only build associative, correlation networks and do not discriminate between direct causal connections and correlations [50,51]. They also do not detect feedback loops. In contrast, MRA infers causal connections, feedback loops, and their strengths, but requires as many perturbations as the number of network modules while being sensible to noise [10,45,52]. To decrease the number of perturbations and improve the MRA tolerance to noise, we developed a Bayesian MRA formulation (BMRA) that also allows the incorporation of existing knowledge and correlation networks as a prior network to improve noise tolerance and inference precision [38,53,54].

### 3.9. Reconstruction of Core Networks in BC and Normal Breast Tissue Derived Cell Lines

To understand critical signaling differences between different cell phenotypes, we reconstructed the quantitative topologies of core networks in these cells. The strengths of causal connections inferred by BMRA depend on the time point at which data have been collected, because the network evolves over time [33]. For a core network consisting mainly of kinases, the early time points where phosphorylation responses are near maximum values reveal the causal signaling connections, whereas the late time points help us understand how the core network affects the DPD modules that specify the cell phenotype. Accordingly, we grouped the time points into two different categories: the early time points (7, 9, 13, and 17 min), and the late time points (40 and 60 min). Consensus networks for the early and the late time points were constructed by concatenating the global response matrices corresponding to different time points. BMRA infers the values of connection coefficients (strengths) between the network nodes together with the confidence intervals (see zip-archive “cSTAR_networks.zip”). The inferred connection strengths also quantify the influence of signaling pathways on the phenotypic cell features, the DPD_onc_ and DPD_L-B_ modules. This enables us to determine the primary and secondary drivers of cell phenotypes. The driver ranks are determined by the inferred local and global influences of signaling pathways on the DPD modules.

### 3.10. Luminal BC Cell Lines

At early time points, BMRA-reconstructed networks give only causal signaling connections, and at the late time points these networks include both signaling connections and the influence of signaling pathways on the phenotypes described by the DPD_onc_ and DPD_L-B_ scores. The results for the late time points suggest that mTOR is a direct, causal oncogenic driver that activates the DPD_onc_ module in almost all 33 luminal BC cell lines (the corresponding matrices of connection coefficients are given in the SI file cSTAR_networks.zip). These findings are in line with clinical reports about the effectiveness of mTOR inhibitors in luminal BC [55,56]. A main causal, direct driver of maintaining the luminal phenotype is STAT3 that negatively changes the DPD_L-B_ score, thereby pushing BC cells to maintain the luminal subtype.

**Figure 2:**
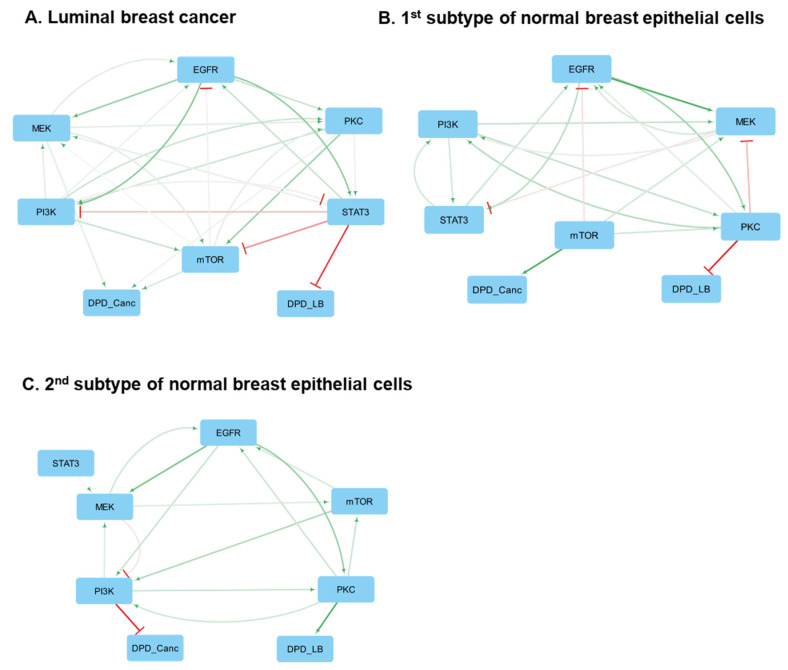
Consensus networks constructed for the late time points and containing both signaling modules and the DPD phenotypic modules. Activation and inhibition are indicated by green arrowheads and red barheads, respectively. The darker the color, the higher the absolute value of the connection coefficient. (**A**) Luminal breast cancer cell lines. The first subtype (**B**) and the second subtype (**C**) of normal breast epithelial derived cell lines.

Although each luminal BC cell line has a specific core network (see SI file cSTAR_networks.zip), a consensus network can still be derived, supporting the general conclusions above (Figure 2A and Supplementary Figure S9A). However, specific distinctions between different cell lines in terms of the connection strengths and feedbacks shape the activity patterns of luminal BC cell signaling networks and, as a result, also influence the cell phenotypes, determined by the DPD_onc_ and DPD_L-B_ scores. Along with mTOR, the MEK/ERK pathway and the STAT3 pathway are critical in activating the DPD_onc_ module in ten and six luminal cell lines, respectively (SI file cSTAR_networks.zip). In agreement with these results, UACC-893 and MDA-MB-453 luminal cell lines [57,58] can be classified as MEK/ERK driven, and the Genomics of Drug Sensitivity in Cancer (GDSC) database suggests that these cell lines are sensitive to ERK inhibitors [59]. Likewise, whereas STAT3 is a main immediate driver of the luminal subtype, in some BC cells, mTOR and MEK/ERK are direct activators of the DPD_L-B_, thus facilitating a transition to the basal-like subtype.

Summarizing, the conclusions derived from the consensus network hold true for luminal BC cell lines. Although the efficiency of drug inhibition of the MEK/ERK, PI3K/AKT, and PKC signaling pathways will depend on the type of cell line, mTOR can serve as a pan-luminal BC target.

### 3.11. Basal BC Cell Lines

**Figure 3:**
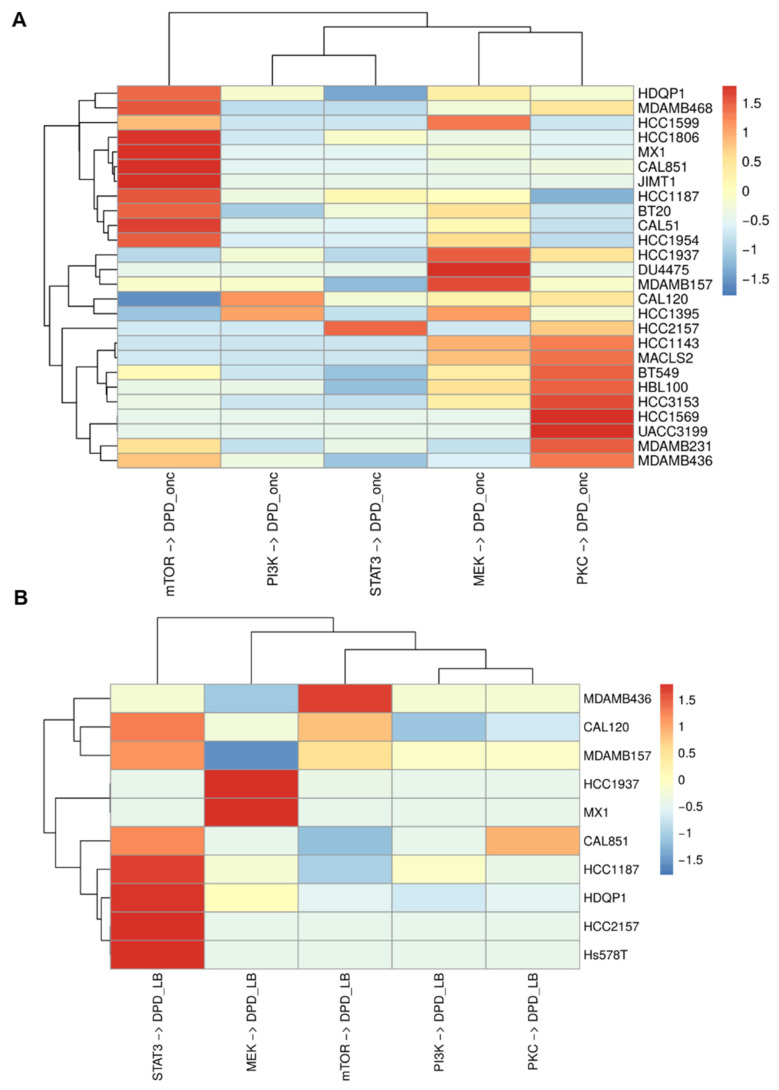
cSTAR-inferred immediate connections from signaling modules to the DPD modules within the core networks basal BC cell lines. (**A**) Connections to the DPD_onc_ module. (**B**) Connections to the DPD_L-B_ module. Only non-zero connections are shown.

Contrary to the possibility to derive a consensus core network for luminal BC cells, the inferred core network circuitries of 28 basal BC cell lines show dramatic differences. Of note, publicly available mass cytometry data for these lines exhibit missing points and show greater variability compared to the data for luminal BC cell lines [6]. Figure 3A exhibits the heatmap that illustrates the BMRA-inferred immediate connections from core network modules to the DPD_onc_. Hierarchical clustering of this heatmap (see dendrograms) suggests that the BMRA-inferred core networks for the analyzed basal BC cell lines can be roughly classified into four distinct groups based on the immediate drivers of their oncogenic transformation. All reconstructed networks are provided in the SI file cSTAR_networks.zip.

In the first group comprising 11 basal BC cell lines (HDQP1, MDAMB468, HCC1599, HCC1806, MX1, CAL851, JIMT1, HCC1187, BT20, CAL51, and HCC1954), mTOR stands out as a main, direct driver of oncogenic transformation. Additionally, similarly to luminal BC, MEK and PKC can assume a secondary role as immediate oncogenic drivers in select cell lines within this group. This suggests that while mTOR serves as the primary drug target for these cells, synergistic two-drug combinations might entail co-targeting of the secondary driver, which varies among the cell lines within this group. For example, MEK is an important oncogenic driver in HCC1599 cells, and PKC is a significant driver in the MDA-MB-468 cell line.

In the second group of five basal BC cell lines (HCC1937, DU4475, MDAMB157, CAL120, and HCC1395), a primary, immediate oncogenic driver is the MEK/ERK pathway, with PKC and PI3K/AKT signaling playing a secondary role. Interestingly, certain cell lines within this group, such as MDA-MB-157, exhibit notable resistance to MEK inhibitors [59]. This can be explained by negative feedback connections within the RAS-RAF-MEK-ERK cascade. These negative feedback mechanisms contribute to the robustness of this cascade against inhibition [60,61]. Hence, to devise synergistic drug combinations for these five basal BC cell lines, additional perturbations targeting various components of the RAS-RAF-MEK-ERK signaling cascade are necessary, which are absent in the existing dataset. 

A third basal BC group consists of only one cell line, HCC2157, whose primary, immediate oncogenic driver is STAT3. The secondary oncogenic driver in this group is PKC.

In the fourth basal BC group comprising nine basal BC cell lines (HCC1143, MACLS2, BT549, HBL100, HCC3153, HCC1569, UACC3199, MDAMB231, and MDAMB436), PKC emerges as primary oncogenic driver. In this group, MEK and mTOR can be secondary drivers in cell-specific fashion, suggesting that PKC inhibitors should be the optimal choice for mono-therapy, while combinations of PKC inhibitors with MEK or mTOR inhibitors could be effective synergistic combinations.

Due to noise in the available data, immediate connections from signaling pathways to the DPD_L-B_ were only identified in 10 basal BC cell lines (Figure 3B). In seven of these cell lines, STAT3 was the primary maintainer of the basal phenotype. In the remaining three cell lines, MEK/ERK and mTOR modules were the primary drivers of the basal phenotype. Interestingly, for BC cells where STAT3 was a primary driver of the basal-like subtype, their driver of oncogenic transformation could be any of mTOR, MEK/ERK, PKC, or STAT3 core network modules (Figure 3A). 

Broadly speaking, basal BC cell lines can be categorized based on the primary drivers of oncogenic transformation (i.e., immediate connections to the DPD_onc_), or the primary drivers of the basal BC subtype (i.e., immediate connections to the DPD_L-B_). These two distinct classifications of basal cell line types appeared to be independent, which can explain inconsistencies in the basal BC subtype classification in the literature [62]. 

### 3.12. Normal Breast Tissue-Derived Cell Lines

The analysis of normal breast tissue cell lines showed significant variability between the inferred core network circuitries, as was also seen for basal BC cell lines. The inferred casual connections partition these cells into two groups with different core network architectures (Figure 2B,C and Supplementary Figure S9B,C). The primary difference between these two groups is the connections from the signaling pathways to the DPD modules. In the first group, whose typical representative is the MCF10A cell line, the mTOR pathway can act as a driver of oncogenic transformation, if strong activation of the mTOR module is sustained for the late time points. In addition to MCF10A cells, activation of the mTOR pathway can potentially transform other cell lines of the first group, i.e., the MCF12A and MCF10F cell lines. Strikingly, both the basal DPD_L-B_ levels (Figure 1B and Supplementary Table S3) and the core network circuitry of MCF10A cells (Figure 2B and the SI file cSTAR_networks.zip) differ dramatically from luminal BC cells, suggesting that following oncogenic transformation these cells will become basal BC type rather than luminal BC type.

The consensus network built for the second group of normal breast tissue cell lines suggests that none of the core network components selected by the available data is a strong oncogenic driver in these cells (Figure 2C, SI file cSTAR_networks.zip). A typical representative of this group is the 184A1 cell line. While these cells are potentially capable of undergoing transformation, the selected components of the core network do not appear to contribute directly to the oncogenic phenotype. These results indicate that the detection of only 29 phosphosites [6] is insufficient for constructing comprehensive core networks using the STVs and for analyzing transformation pathways, using the DPDs determined by such a limited number of cellular molecular features. At the same time, the cells in the second group retain some common signaling motifs also observed in BC cells, such as positive feedback loops between MEK/ERK and EGFR.

### 3.13. MRA Predicts Phenotypic Effects of Drug Responses That Correlate with Available Data

Forward MRA formalism allows us to predict the global BC cell responses to drug perturbations of core network pathways [39,60,63,64]. We express these BC cell responses to drugs in terms of the resulting changes in the $DPD_{onc}$ scores, which quantify the phenotypic status of oncogenic transformation. Mathematically, the global responses ($R_{xI}$) of all network modules ($x=x_{1},\;\ldots ,x_{n}$) that encompass both signaling pathways and phenotypic modules to a drug (*I*) are obtained using the inversion of the BMRA-inferred local response matrix (r) and a vector ($r_{xI}$) of the local responses of the primary targets to this drug [10,60]. This relationship is expressed as follows:

(10) $$R_{xI}=-r^{-1}\cdot r_{xI}$$

From Equation (10) it follows, that if a signaling module (*j*) is inhibited by 1%, the *j*-th column of the matrix $r^{-1}$ represents the global responses of each network module to this inhibition. In particular, using the matrix $r^{-1}$ we can predict the changes in the oncogenic transformation status, quantified by the $DPD_{onc}$ scores following drug inhibition of each core network signaling pathway. To compare these predictions with available literature data, we collected the IC50 viability data for basal BC cell lines from the PharmacoDB database [65]. When multiple IC50 values for a drug were provided, we calculated their mean values and analyzed if there was a correlation between BMRA-predicted global responses of the $DPD_{onc}$ to inhibition of EGFR, PI3K, MEK/ERK, and PKC modules with 1/IC50 values. The values 1/IC50 represent sensitivity of cells to inhibition of the appropriate node, so we expected a positive correlation between global responses of $DPD_{onc}$ and 1/IC50. We did not include the mTOR module in this analysis, since the data for the mTOR inhibitor used in the original study, Sirolimus [8], were absent in PharmacoDB for most basal breast cancer cell lines.

We found that the global responses of $DPD_{onc}$ correlate reasonably well with 1/IC50 values with positive correlation coefficients, as expected (Supplementary Figure S10). This analysis suggests that even straightforward MRA-based models (Equation (10)) demonstrate significant predictive power, as supported by existing literature data.

### 3.14. Building cSTAR Models: Digital Cell Twins

As a next step, we aimed to develop nonlinear models to predict phenotypic transitions of breast cancer cell lines at a larger scale. Predicting the effectiveness of drug combinations requires non-linear mechanistic models. Only these models, referred to as digital cell twins, can predict whether drug treatments are synergistic, antagonistic, or merely additive [66]. 

The topology of core controlling networks is quantified in terms of the connection coefficients (SI file cSTAR_networks.zip), which are closely related to the elements of the Jacobian matrix of the underlying dynamical system [10,67]. Therefore, the precise inference of casual network connections paves the way to building mechanistic dynamical models of core controlling networks and how they influence cell phenotypes [5,35,54]. These cSTAR models explain and predict how diverse experimental manipulations, including drug treatments, alter signaling patterns and cell states. We posit these models are digital cell twins, whose outputs predict how cells travel through Waddington’s landscape, following internal and external cues and perturbations [33]. We generate these landscapes in the DPD_onc_ and DPD_L-B_ space [35]. 

Here, we focus on basal BC cell lines, because of the unmet clinical need for novel, synergistic treatments of basal BCs. These models must be cell line-specific, and we build them for typical representatives of the three groups of basal BC cell lines found to have different oncogenic drivers. A description of the model derivation and parameter estimation is given in Section 2 (see Section 2.4, Section 2.5 and Section 2.6). Experimental and simulated time courses of phosphorylated MKK4, AKT, GSK3β, RSK, STAT3, and RPS6 are shown in Supplementary Figures S2–S4 for certain basal BC cell groups.

### 3.15. cSTAR Model of the MDA-MB-468 Cell Line Suggests Experimental Interventions Normalizing Phosphoproteomic Patterns of the First Group of Basal BC Cells

**Figure 4:**
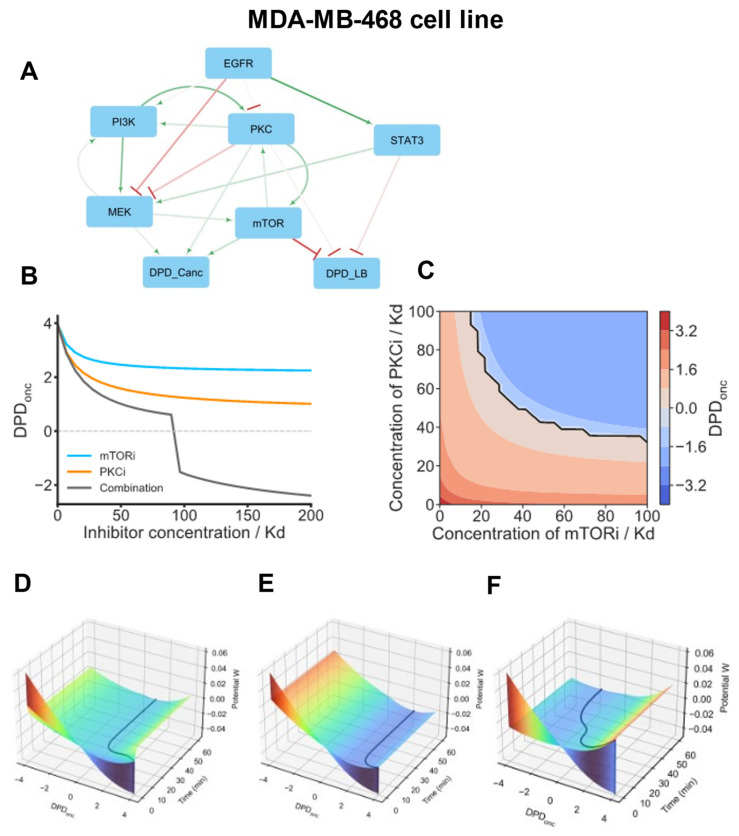
cSTAR model of the MDA-MB-468 cell line. (**A**) BMRA-reconstructed topology of the core signaling network and the connections to the DPD modules at the late time points for the MDA-MB-468 cell line. Activation and inhibition are indicated by green arrowheads and red barheads, respectively. The darker the color, the higher the absolute value of the connection coefficient. (**B**) Dose responses of the DPD_onc_ scores to mTOR, PKC inhibitors (mTORi, PKCi), and their combination. In the drug combination, the ratio of mTORi and PKCi doses is 1:1, with each inhibitor dose being 50% of the corresponding dose when the inhibitors are applied separately. (**C**) Predictive simulations of the Loewe isoboles demonstrate synergy in a combination of mTORi and PKCi. Solid black line represents a borderline between normal and oncogenic states, i.e., DPD_onc_ = 0. (**D**–**F**) Model-predicted maneuvering of the MDA-MB-468 cell in Waddington’s landscape (shown by black lines). Surface colors represent the values of Waddington’s potential. After model variables reached the equilibrium, the following inhibitor doses were added at t = 0 individually or in a combination; 100 Kd PKCi (**D**), 100 Kd mTORi (**E**), or 50 Kd PKCi + 50 Kd mTORi (**F**). (**D**,**E**) PKCi and mTORi applied as single drug treatments do not normalize the phosphoproteomic cell patterns. (**F**) By combining these inhibitors in twice lower doses, the DPD_onc_ trajectory shows a switch from positive DPD_onc_ scores to negative DPD_onc_ scores, which correspond to non-malignant breast tissue cells.

For the first basal subtype group, we focus on modeling the MDA-MB-468 cell line. The core network inferred for this cell line suggests that mTOR is the main oncogenic driver in these cells, with PKC being a secondary driver, and MEK/ERK only weakly contributing to DPD_onc_ (Figure 4A and SI file cSTAR_networks.zip). Importantly, PKC and mTOR form a mutual activation loop. Thus, being activated by EGFR-PI3K signaling, the mTOR-PKC pair self-amplifies its signaling, generating sustained activating signals for oncogenic transformation.

Our cSTAR model suggests that co-inhibition of mTOR and PKC can disrupt this vicious activation loop and normalize the phosphoproteomic patterns of the MDA-MB-468 cells, moving these closer to the patterns of normal breast tissue-derived cells. Figure 4B,C show dose responses and Loewe isoboles for DPD_onc_, confirming that a combination of mTORi and PKCi synergistically suppresses the DPD_onc_, pushing the cells closer to a normal phenotype (Loewe isoboles are straight lines for non-interacting drugs, concave for synergistic drugs and convex for drugs that antagonize).

cSTAR models enable the reconstruction of Waddington’s landscape and suggest experimental perturbations that control cell state transitions [5]. To further investigate why MDA-MD-468 cells are resistant to mTORi and PKCi applied separately, while a combination of these inhibitors normalizes the DPD_onc_ score, we reconstructed Waddington’s landscape and compared cell trajectories (Figure 4D–F). These trajectories showed that when mTORi or PKC are used on their own, MDA-MD-468 cells reside in a stable steady state that corresponds to an oncogenic state and cannot overcome the barrier to escape from this state. However, a combination of PKCi and mTORi alters the shape of Waddington’s landscape and enables cells to traverse through the barrier separating the MDA-MB-468 cell states that correspond to oncogenic vs. normal DPD_onc_ scores. The abrupt decrease in the DPD_onc_ score (Figure 4F) is explained by a saddle-node bifurcation (a fold catastrophe) that occurs when a stable steady-state solution corresponding to the oncogenic state disappears [68].

### 3.16. STAT3-Driven Basal BC Cells

**Figure 5:**
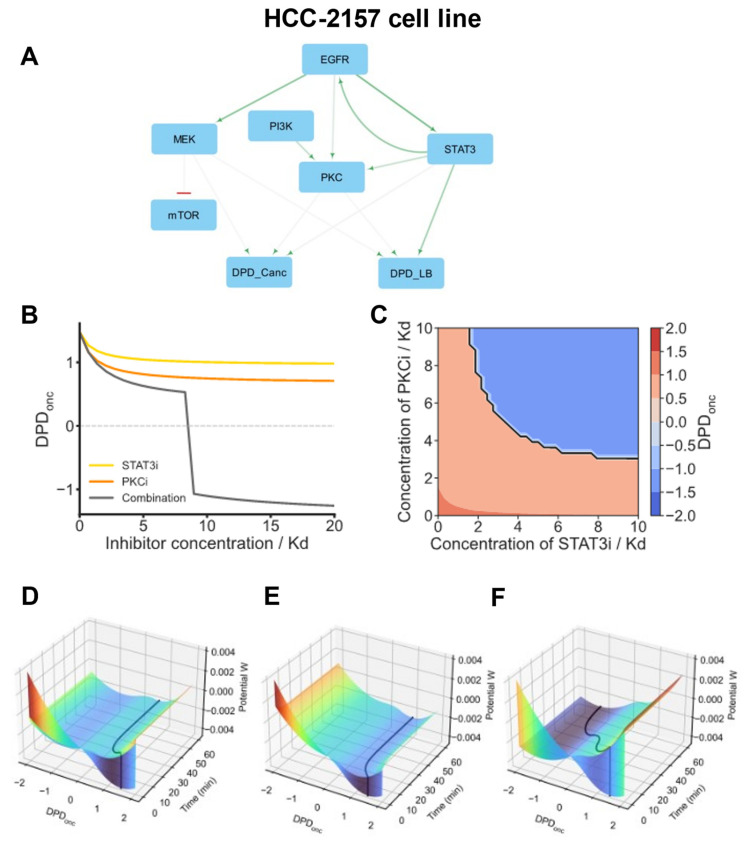
cSTAR model of the HCC-2157 cell line. (**A**) BMRA-reconstructed topology of the core signaling network and the connections to the DPD modules at the late time points for the HCC-2157 cell line. Activation and inhibition are indicated by green arrowheads and red barheads, respectively. The darker the color, the higher the absolute value of the connection coefficient. (**B**) Dose responses of the DPD_onc_ scores to STAT3i, PKCi, and their combination. In the drug combination, the ratio of STAT3i and PKCi doses is 1:1, with each inhibitor dose being 50% of the corresponding dose when the inhibitors are applied individually. (**C**) Steady-state DPD_onc_ responses to PKCi and STAT3i were analysed using the Loewe isoboles. Solid black line represents a borderline between normal and oncogenic states, i.e., DPD_onc_ = 0. The concave isoboles demonstrate that these inhibitors have a synergistic effect on normalizing phosphoproteomic patterns. (**D**–**F**) Model-predicted maneuvering of the HCC-2157 cells in Waddington’s landscape (shown by black lines). After model variables reached the equilibrium, the following inhibitor doses were added at t = 0, separately and in a combination; 16 Kd PKCi (**D**), 16 Kd STAT3i (**E**), or 8 Kd PKCi + 8 Kd STAT3i (**F**). STAT3i and PKCi applied as single drug treatments do not normalize the phosphoproteomic cell patterns (**D**,**E**). The STAT3i and PKCi combination in twice lower doses normalizes the DPD_onc_ scores of the HCC-2157 cells (**F**).

Next, we analyze the HCC2157 cell line, which itself forms a distinct group separate from other basal cell line groups. For this cell line, the primary immediate driver of both oncogenic transformation and basal-like subtype is STAT3. STAT3 is robustly activated by EGFR, and this activation is sustained due to strong positive feedback from STAT3 to EGFR (Figure 5A). Additionally, EGFR activates PI3K, which in turn activates PKC. PKC is the second oncogenic driver in these cells, and there is a positive feedback loop between PKC and STAT3 observed at the early time points (SI file cSTAR_networks.zip). The resulting amplification of STAT3 and PKC signals drives the oncogenic state of HCC2157 cells. As a result, these cells are sensitive to EGFR inhibitors and resistant to MEK and ERK inhibitors, which agrees with the experimental data [59]. According to our model, the optimal two-drug combination for this cell line is a pairing of STAT3 and PKC inhibitors. (Figure 5B,C). A combination of STAT3i and PKCi triggers cell state transition from oncogenic to normal DPD_onc_ scores, whereas single treatments by individual drugs at twice higher concentrations cannot move the DPD_onc_ scores to the DPD_onc_ range of normal breast tissue-derived cell lines. The model-based predictions are further supported by simulating HCC-2157 cell maneuvering in Waddington’s landscape following inhibitor treatments. Figure 5D–F demonstrate that the normalization of cell state, i.e., a transition of the DPD_onc_ scores from positive to negative values, can only happen when STAT3i and PKCi are used in combination, and remarkably at twice lower doses than each inhibitor on its own.

### 3.17. cSTAR Model of the MDA-MB-231 Cell Line of the Fourth Basal BC Cell Group

**Figure 6:**
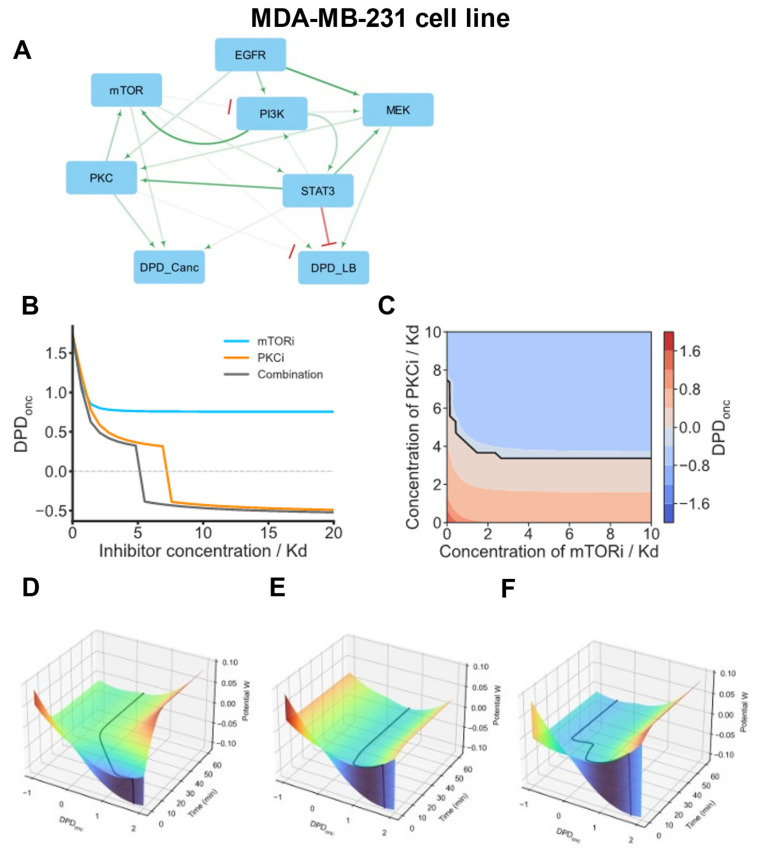
cSTAR model of the MDA-MB-231 cell line. (**A**) BMRA-reconstructed topology of the core signaling network and the connections to the DPD modules at the late time points for the MDA-MB-231 cell line. Activation and inhibition are indicated by green arrowheads and red barheads, respectively. The darker the color, the higher the absolute value of the connection coefficient. (**B**) Dose responses of the DPDonc to mTORi, PKCi, and their combination. The ratio of mTORi and PKCi doses in a combination is 1:2, and their doses equal 33% and 67% of the corresponding inhibitor dose when inhibitors are applied separately. (**C**) Predictive simulations of the Loewe isoboles demonstrate synergy by the combination of mTORi and PKCi. Solid black line represents a borderline between normal and oncogenic states, i.e., DPDonc = 0. (**D**–**F**) Model-predicted maneuvering of the MDA-MB-231 cells in Waddington’s landscape (shown by black lines). Color represents the value of Waddington’s landscape potential. After model variables reached the equilibrium, the following inhibitor doses were added at t = 0 separately or in a combination; 7 Kd PKCi (**D**), 10 Kd mTORi (**E**), or 3.5 Kd PKCi + 5 Kd mTORi (F). (**D**,**E**) Cells treated with moderate mTORi doses or large PKCi doses applied separately remain in the malignant DPD_onc_ score area. (**F**) By combining these inhibitors in twice lower doses, the DPD_onc_ trajectory shows a switch from positive to negative DPD_onc_ scores, corresponding to non-malignant breast tissue cells.

The oncogenic status of the fourth basal BC cell group, including its typical representative, the MDA-MB-231 cell line, is mainly driven by the PKC module, with mTOR and STAT3 playing secondary roles (Figure 6A). In addition to its immediate effects on the DPD_onc_, the PKC module also activates mTOR. Other pathways and signaling hubs, such as MEK/ERK and PI3K/AKT, operate as oncogenic drivers by activating the mTOR and PKC modules. A cSTAR model suggests that a PKC inhibitor is the most efficient single drug to normalize the DPD_onc_ scores, whereas mTOR inhibition is less effective (Figure 6B). Further, the model predicts that the optimal two-drug combinations are combinations that inhibit two immediate oncogenic drivers, mTOR and PKC. Importantly, a combination of mTORi and PKCi is synergistic, as demonstrated by concave Loewe isoboles in Figure 6C. This synergy enables a substantial decrease in the doses of inhibitor treatment [66].

The emergence of resistance to targeted therapies is often related to rewiring network connections, including pathway crosstalk and feedback loops. For instance, the positive influence of the PI3K/AKT pathway on the MEK/ERK pathway (Figure 6A) may change after inhibition, as observed in many cell systems [69]. Therefore, although the synergy scores of model-suggested alternative target combinations are comparable, co-inhibition of the mTOR and PKC modules appears to be the best combination, as long as these modules continue to be direct oncogenic drives that increase the DPD_onc_ score. A digital twin model of the MDA-MB-231 cell line predicts how these cells traverse Waddington’s landscape following inhibitor treatments (Figure 6D–F). Even large mTORi doses cannot move the MDA-MB-231 cell state from oncogenic to normal DPD_onc_ scores, whereas high PKCi doses can. However, a combination of PKCi and mTORi at twice lower doses switches MDA-MB-231 cells from oncogenic to normal DPD_onc_ scores. This abrupt switch corresponds to a saddle-node bifurcation (Figure 6B,F).

## 4. Discussion

In previous work we showed that cSTAR can accurately detect cell states and devise precise interventions to convert undesired into desired cell states [5,36]. This is achieved through cSTAR capabilities to extract dynamic mechanistic models from omics data using perturbation approaches [70]. This property makes cSTAR recommendations fully tractable and explainable, which is critical for many biomedical applications including the development of digital twins [71]. Thus, it is important to evaluate which data types cSTAR can use and how well it can cope with the sparseness and noisiness of biological and biomedical data. With this aim in mind, we analyzed a CYTOF data set that had proven challenging in a previous analysis due to these constraints [6]. We showed that cSTAR copes with these challenges by providing a classification of both the BC and non-malignant breast tissue cell lines, while also suggesting interventions to normalize DPD scores. Exploring the limits of molecular features and perturbations necessary for cSTAR to work reliably, we found that a limited number of molecular features and perturbations reported in Ref. [6] suffice to produce robust results. Using this CYTOF dataset [6], we were able to apply the cSTAR pipeline to map cell states, determine cell-specific networks that control cell state transitions, and build digital twin cell models that predicted targeted therapeutic interventions to normalize phosphoproteomic patterns of luminal and distinct basal BC subtypes. Ultimately, these predictions must be tested against the experiment. Because our own experimental verification was beyond the scope of this work, we computationally demonstrated robustness of cSTAR predictions and reported clinical and biochemical data that support them. 

Gene mutations and other genetic aberrations embedded in individual protein expression landscapes drive pathological BC phenotypes through deregulated input–output behaviors of complex molecular networks. Therapeutic strategies to inhibit a mutated or overexpressed oncogene rarely work as anticipated, because of combinatorial molecular interactions leading to the activation of alternative signaling pathways. New approaches to understanding the causes of intrinsic and acquired drug resistance to targeted therapies are needed, that include the dynamic understanding of cell regulatory network adaptations in which the targets are embedded.

Deliberately engineering cell phenotypes is a long-standing problem in biology [72,73]. Waddington’s visionary introduction of a landscape where cells are symbolized by marbles that roll into different valleys that represent different cell states [7] offers an intuitive visualization that over six decades has proven to be a useful framework for studies in developmental biology and cancer biology [74,75]. We have made significant progress in understanding why and how cells choose certain pathways through Waddington’s landscape [75] and being able to control these decisions would open unprecedented possibilities for fields where the precise control of cell fate decisions is paramount for success. These include biotechnology, tissue engineering, and treatment of diseases that involve tissue remodeling and re-/dedifferentiation, such as cancer and chronic inflammatory diseases.

A remarkable example showing that the control of cell phenotypes and fate decisions can be achieved is the reprogramming of differentiated somatic cells into induced pluripotent stem cells (iPSCs) by the transfer of selected genes [73]. In Waddington’s terms iPSCs are a reset of the cell fate tree to the origin. For many applications a more subtle control of cell fates within the Waddington landscape would be preferable. Designing such approaches has been hampered by the complex and dynamic nature of the signaling pathways that control cell fate decisions [76]. 

Our manuscript encompasses both our perspective on the key challenges of 21st-century biology and a computational study utilizing the innovative cSTAR approach. This study yields cell phenotype predictions that are challenging to obtain through current bioinformatics methods. It is important to emphasize that the literature corroborates many of our conclusions, e.g., responses of the DPD_onc_ to single drugs are in line with the GDSC data [59]. At the same time, certain, novel cSTAR predictions have not yet been examined by other research groups. Nevertheless, indirect evidence supports the predicted mTORi and PKCi drug combinations, which have previously demonstrated their effectiveness in a distinct cancer cell context [77]. 

At the same time, given the published CYTOF dataset [6], there are two unavoidable limitations. The first is that the phosphoproteomic responses were measured only up to one hour. Thus, the phenotypic changes that require more time to be observed could be masked. Second, only a small number of antibodies were used, and measuring 29 analytes is borderline for the applicability of cSTAR. For example, the extraction of core network components almost did not affect the precision of classification of cancer vs non-cancer cell lines, but it did affect the precision of classification of BC subtype. To step beyond available perturbation CYTOF data and to understand how big the number of analytes measured must be to capture cell state transitions, we utilized proteomic data for the same cell lines (see Section 3.4 and Supplementary Figure S8). We found that if analytes are selected randomly, ca., 300 analytes must be measured to achieve classification precision of more than 90%. However, if the choice of analytes to measure is supervised, even with 10 analytes the flawless classification of cell states can be achieved. Thus, application of cSTAR requires either almost whole-proteome perturbation measurements, such as mass spectrometry phosphoproteomics, or careful supervised choice of ca. 30–100 analytes to be measured, which can be achieved by CYTOF, reverse phase protein arrays or similar techniques.

In summary, we showed that cSTAR can generate digital twins, i.e., actionable computer models of a human cell. The cSTAR digital cell twin models were developed using a combination of machine learning and dynamical modeling approaches coupled with the reconstruction of causal connections in core regulatory networks that control cell fates. These models are hybrid models, i.e., models that combine elements of artificial intelligence with elements of mechanistic physics-based modeling. The ever-increasing pace of acquiring omics data using systematic perturbations will allow the design of precise approaches to direct purposefully cancer cell states to physiological states. As such data are increasingly being produced on cancer patients, we envision that our approach will facilitate the generation of digital twin models of individual cancer patients. Such models could provide clinicians with a useful tool for supporting their clinical treatment decisions, including the choice of drugs and their combinations. 

## 5. Conclusions

We exploited a cSTAR approach to analyze a mass cytometry dataset comprising 62 BC and five normal breast tissue cell lines, each subjected to five distinct kinase inhibitor perturbations [6]. In the molecular data space, using cSTAR we precisely separated normal vs cancer cell states, as well as luminal vs basal BC cells, aimed to elucidate the molecular mechanisms of oncogenic transformation and identify potential reversal strategies. Although a limited number of 29 analytes constrained the cSTAR prediction accuracy, we could distinguish robustly between molecular mechanisms that drove oncogenic transformation in luminal and basal BC cells. cSTAR analysis revealed that luminal BC cell lines exhibit more uniform signaling networks compared to basal BC cell lines. In luminal BC, mTOR consistently emerged as the primary oncogenic driver, while the PKC and MEK/ERK pathways played secondary roles. For basal BC cell lines, we identified four distinct groups whose oncogenic states were primarily driven by (i) mTOR, (ii) MEK/ERK, (iii) STAT3, and (iv) PKC pathways. For most of these groups, cSTAR computational analysis suggested synergistic drug combinations targeting primary oncogenic drivers and secondary pathways, which varied among BC cells within each group. While these predictions align with the existing literature, biochemical, and clinical data, conducting more precise experimental validations remains an attractive goal for future research. 

## Data Availability

The SBML format cell line-specific models and the inferred connection strengths of the core network are available at Supplementary Materials (Models.zip and cSTAR_networks.zip, respectively).

## Supplementary Materials

The following supporting information can be downloaded at: https://www.mdpi.com/article/10.3390/cancers16132354/s1, Figure S1: Schematic of the model refinement: the EGFR/MAPK pathway activation; Figure S2: cSTAR model recapitulates the signaling and DPD dynamics of the MDA-MB-468 cell line; Figure S3: Model recapitulates signalling and DPD dynamics in HCC-2157 cell line; Figure S4: Model recapitulates signalling and DPD dynamics in MDA-MB-231 cell line; Figure S5: Hierarchical clustering analysis of the averaged values of CYTOF data is unable to separate cell states; Figure S6: Removal of a single analyte from the CYTOF dataset does not affect the classification of cell states by cSTAR; Figure S7: Robustness of the cell state classification with respect to noise; Figure S8: The quantity and specificity of the analytes are pivotal factors for accurate cell state classification; Figure S9: Consensus signaling networks reconstructed for the early time points; Figure S10: Model-predicted drug responses at the systems level correlate with the drug sensitivities of basal BC cell lines; Table S1: CYTOF data after normalization and pre-processing; Table S2: Components of the STV vectors; Table S3: DPD scores for all cell lines; Table S4: Module outputs and perturbations used for BMRA network reconstruction; Table S5: Prior network topology; Table S6: List of proteins used in Figure S8C.

## References

[1] La Manno G., Soldatov R., Zeisel A., Braun E., Hochgerner H., Petukhov V., Lidschreiber K., Kastriti M.E., Lönnerberg P., Furlan A. (2018). RNA Velocity of Single Cells. Nature.

[2] Schiebinger G., Shu J., Tabaka M., Cleary B., Subramanian V., Solomon A., Gould J., Liu S., Lin S., Berube P. (2019). Optimal-Transport Analysis of Single-Cell Gene Expression Identifies Developmental Trajectories in Reprogramming. Cell.

[3] Qiu X., Zhang Y., Martin-Rufino J.D., Weng C., Hosseinzadeh S., Yang D., Pogson A.N., Hein M.Y., Hoi (Joseph) Min K., Wang L. (2022). Mapping Transcriptomic Vector Fields of Single Cells. Cell.

[4] Yeo G.H.T., Saksena S.D., Gifford D.K. (2021). Generative Modeling of Single-Cell Time Series with PRESCIENT Enables Prediction of Cell Trajectories with Interventions. Nat. Commun..

[5] Rukhlenko O.S., Halasz M., Rauch N., Zhernovkov V., Prince T., Wynne K., Maher S., Kashdan E., MacLeod K., Carragher N.O. (2022). Control of Cell State Transitions. Nature.

[6] Tognetti M., Gabor A., Yang M., Cappelletti V., Windhager J., Rueda O.M., Charmpi K., Esmaeilishirazifard E., Bruna A., de Souza N. (2021). Deciphering the Signaling Network of Breast Cancer Improves Drug Sensitivity Prediction. Cell Syst..

[7] Waddington C.H. (1940). Organisers and Genes.

[8] Tognetti M., Gabor A., Yang M., Cappelletti V., Windhager J., Rueda O.M., Charmpi K., Esmaeilishirazifard E., Bruna A., de Souza N. (2021). Deciphering the Signaling Network Landscape of Breast Cancer Improves Drug Sensitivity Prediction. Mendeley Data.

[9] Lill D., Rukhlenko O.S., Mc Elwee A.J., Kashdan E., Timmer J., Kholodenko B.N. (2019). Mapping Connections in Signaling Networks with Ambiguous Modularity. NPJ Syst. Biol. Appl..

[10] Kholodenko B.N., Kiyatkin A., Bruggeman F.J., Sontag E., Westerhoff H.V., Hoek J.B. (2002). Untangling the Wires: A Strategy to Trace Functional Interactions in Signaling and Gene Networks. Proc. Natl. Acad. Sci. USA.

[11] Lopez C.F., Muhlich J.L., Bachman J.A., Sorger P.K. (2013). Programming Biological Models in Python Using PySB. Mol. Syst. Biol..

[12] Tsyganov M.A., Kolch W., Kholodenko B.N. (2012). The Topology Design Principles That Determine the Spatiotemporal Dynamics of G-Protein Cascades. Mol. Biosyst..

[13] Storn R., Price K. (1997). Differential Evolution—A Simple and Efficient Heuristic for Global Optimization over Continuous Spaces. J. Glob. Optim..

[14] Virtanen P., Gommers R., Oliphant T.E., Haberland M., Reddy T., Cournapeau D., Burovski E., Peterson P., Weckesser W., Bright J. (2020). SciPy 1.0: Fundamental Algorithms for Scientific Computing in Python. Nat. Methods.

[15] Hunter J.D. (2007). Matplotlib: A 2D Graphics Environment. Comput. Sci. Eng..

[16] Waskom M. (2021). Seaborn: Statistical Data Visualization. J. Open Source Softw..

[17] Plischke M., Bergersen B. (2006). Equilibrium Statistical Physics.

[18] Koboldt D.C., Fulton R.S., McLellan M.D., Schmidt H., Kalicki-Veizer J., McMichael J.F., Fulton L.L., Dooling D.J., Ding L., Mardis E.R. (2012). Comprehensive Molecular Portraits of Human Breast Tumours. Nature.

[19] Curtis C., Shah S.P., Chin S.F., Turashvili G., Rueda O.M., Dunning M.J., Speed D., Lynch A.G., Samarajiwa S., Yuan Y. (2012). The Genomic and Transcriptomic Architecture of 2000 Breast Tumours Reveals Novel Subgroups. Nature.

[20] Chin K., DeVries S., Fridlyand J., Spellman P.T., Roydasgupta R., Kuo W.-L., Lapuk A., Neve R.M., Qian Z., Ryder T. (2006). Genomic and Transcriptional Aberrations Linked to Breast Cancer Pathophysiologies. Cancer Cell.

[21] Porta-Pardo E., Valencia A., Godzik A. (2020). Understanding Oncogenicity of Cancer Driver Genes and Mutations in the Cancer Genomics Era. FEBS Lett..

[22] Martincorena I., Campbell P.J. (2015). Somatic Mutation in Cancer and Normal Cells. Science.

[23] Magwene P.M., Lizardi P., Kim J. (2003). Reconstructing the Temporal Ordering of Biological Samples Using Microarray Data. Bioinformatics.

[24] Cacchiarelli D., Qiu X., Srivatsan S., Manfredi A., Ziller M., Overbey E., Grimaldi A., Grimsby J., Pokharel P., Livak K.J. (2018). Aligning Single-Cell Developmental and Reprogramming Trajectories Identifies Molecular Determinants of Myogenic Reprogramming Outcome. Cell Syst..

[25] Murtagh F., Contreras P. (2012). Algorithms for Hierarchical Clustering: An Overview. Wiley Interdiscip. Rev. Data Min. Knowl. Discov..

[26] Comon P., Jutten C. (2010). Handbook of Blind Source Separation: Independent Component Analysis and Applications.

[27] Gordonov S., Hwang M.K., Wells A., Gertler F.B., Lauffenburger D.A., Bathe M. (2016). Time Series Modeling of Live-Cell Shape Dynamics for Image-Based Phenotypic Profiling. Integr. Biol..

[28] Cook D.P., Vanderhyden B.C. (2020). Context Specificity of the EMT Transcriptional Response. Nat. Commun..

[29] Breiman L. (2001). Random Forests. Mach. Learn..

[30] Cortez P. (2010). Data Mining with Neural Networks and Support Vector Machines Using the R/Rminer Tool. Advances in Data Mining: Applications and Theoretical Aspects.

[31] Bzdok D., Krzywinski M., Altman N. (2018). Points of Significance: Machine Learning: Supervised Methods. Nat. Methods.

[32] Kolch W., Halasz M., Granovskaya M., Kholodenko B.N. (2015). The Dynamic Control of Signal Transduction Networks in Cancer Cells. Nat. Rev. Cancer.

[33] Hastie T., Tibshirani R., Friedman J.H., Friedman J.H. (2009). The Elements of Statistical Learning: Data Mining, Inference, and Prediction.

[34] Cortes C., Vapnik V. (1995). Support-Vector Networks. Mach. Learn..

[35] Kholodenko B.N., Kolch W., Rukhlenko O.S. (2023). Reversing Pathological Cell States: The Road Less Travelled Can Extend the Therapeutic Horizon. Trends Cell Biol..

[36] Yabaji S.M., Rukhlenko O.S., Chatterjee S., Bhattacharya B., Wood E., Kasaikina M., Kholodenko B.N., Gimelbrant A.A., Kramnik I. (2023). Cell State Transition Analysis Identifies Interventions That Improve Control of Mycobacterium Tuberculosis Infection by Susceptible Macrophages. Sci. Adv..

[37] Haken H. (2004). Synergetics Introduction and Advanced Topics.

[38] Santra T., Rukhlenko O., Zhernovkov V., Kholodenko B.N. (2018). Reconstructing Static and Dynamic Models of Signaling Pathways Using Modular Response Analysis. Curr. Opin. Syst. Biol..

[39] Kholodenko B.N., Westerhoff H.V. (1995). The Macroworld versus the Microworld of Biochemical Regulation and Control. Trends Biochem. Sci..

[40] Peterson A.F., Ingram K., Huang E.J., Parksong J., McKenney C., Bever G.S., Regot S. (2022). Systematic Analysis of the MAPK Signaling Network Reveals MAP3K-Driven Control of Cell Fate. Cell Syst..

[41] Hanson R.L., Batchelor E. (2022). Coordination of MAPK and P53 Dynamics in the Cellular Responses to DNA Damage and Oxidative Stress. Mol. Syst. Biol..

[42] Santos S.D.M., Verveer P.J., Bastiaens P.I.H. (2007). Growth Factor-Induced MAPK Network Topology Shapes Erk Response Determining PC-12 Cell Fate. Nat. Cell Biol..

[43] Nakakuki T., Birtwistle M.R., Saeki Y., Yumoto N., Ide K., Nagashima T., Brusch L., Ogunnaike B.A., Okada-Hatakeyama M., Kholodenko B.N. (2010). Ligand-Specific c-Fos Expression Emerges from the Spatiotemporal Control of ErbB Network Dynamics. Cell.

[44] Kholodenko B.N., Hoek J.B., Westerhoff H.V., Brown G.C. (1997). Quantification of Information Transfer via Cellular Signal Transduction Pathways. FEBS Lett..

[45] Sontag E., Kiyatkin A., Kholodenko B.N. (2004). Inferring Dynamic Architecture of Cellular Networks Using Time Series of Gene Expression, Protein and Metabolite Data. Bioinformatics.

[46] Yalamanchili N., Zak D.E., Ogunnaike B.A., Schwaber J.S., Kriete A., Kholodenko B.N. (2006). Quantifying Gene Network Connectivity In Silico: Scalability and Accuracy of a Modular Approach. Syst. Biol..

[47] Bastiaens P., Birtwistle M.R., Blüthgen N., Bruggeman F.J., Cho K.H., Cosentino C., De La Fuente A., Hoek J.B., Kiyatkin A., Klamt S. (2015). Silence on the Relevant Literature and Errors in Implementation. Nat. Biotechnol..

[48] Kholodenko B.N. (2007). Untangling the Signalling Wires. Nat. Cell Biol..

[49] Mekedem M., Ravel P., Colinge J. (2022). Application of Modular Response Analysis to Medium- to Large-Size Biological Systems. PLoS Comput. Biol..

[50] Huynh-Thu V.A., Irrthum A., Wehenkel L., Geurts P. (2010). Inferring Regulatory Networks from Expression Data Using Tree-Based Methods. PLoS ONE.

[51] Margolin A.A., Nemenman I., Basso K., Wiggins C., Stolovitzky G., Favera R.D., Califano A. (2006). ARACNE: An Algorithm for the Reconstruction of Gene Regulatory Networks in a Mammalian Cellular Context. BMC Bioinform..

[52] Thomaseth C., Fey D., Santra T., Rukhlenko O.S., Radde N.E., Kholodenko B.N. (2018). Impact of Measurement Noise, Experimental Design, and Estimation Methods on Modular Response Analysis Based Network Reconstruction. Sci. Rep..

[53] Santra T., Kolch W., Kholodenko B.N. (2013). Integrating Bayesian Variable Selection with Modular Response Analysis to Infer Biochemical Network Topology. BMC Syst. Biol..

[54] Halasz M., Kholodenko B.N., Kolch W., Santra T. (2016). Integrating Network Reconstruction with Mechanistic Modeling to Predict Cancer Therapies. Sci. Signal..

[55] Rodriguez M.J., Perrone M.C., Riggio M., Palafox M., Salinas V., Elia A., Salgueiro N.D., Werbach A.E., Marks M.P., Kauffman M.A. (2023). Targeting MTOR to Overcome Resistance to Hormone and CDK4/6 Inhibitors in ER-Positive Breast Cancer Models. Sci. Rep..

[56] Rotundo M.S., Galeano T., Tassone P., Tagliaferri P. (2016). MTOR Inhibitors, a New Era for Metastatic Luminal HER2-Negative Breast Cancer? A Systematic Review and a Meta-Analysis of Randomized Trials. Oncotarget.

[57] Hollestelle A., Nagel J.H.A., Smid M., Lam S., Elstrodt F., Wasielewski M., Ng S.S., French P.J., Peeters J.K., Rozendaal M.J. (2010). Distinct Gene Mutation Profiles among Luminal-Type and Basal-Type Breast Cancer Cell Lines. Breast Cancer Res. Treat..

[58] Dai X., Cheng H., Bai Z., Li J. (2017). Breast Cancer Cell Line Classification and Its Relevance with Breast Tumor Subtyping. J. Cancer.

[59] Yang W., Soares J., Greninger P., Edelman E.J., Lightfoot H., Forbes S., Bindal N., Beare D., Smith J.A., Thompson I.R. (2013). Genomics of Drug Sensitivity in Cancer (GDSC): A Resource for Therapeutic Biomarker Discovery in Cancer Cells. Nucleic Acids Res..

[60] Kholodenko B.N., Rauch N., Kolch W., Rukhlenko O.S. (2021). A Systematic Analysis of Signaling Reactivation and Drug Resistance. Cell Rep..

[61] Sturm O.E., Orton R., Grindlay J., Birtwistle M., Vyshemirsky V., Gilbert D., Calder M., Pitt A., Kholodenko B., Kolch W. (2010). The Mammalian MAPK/ERK Pathway Exhibits Properties of a Negative Feedback Amplifier. Sci. Signal..

[62] Neve R.M., Chin K., Fridlyand J., Yeh J., Baehner F.L., Fevr T., Clark L., Bayani N., Coppe J.P., Tong F. (2006). A Collection of Breast Cancer Cell Lines for the Study of Functionally Distinct Cancer Subtypes. Cancer Cell.

[63] Kholodenko B.N., Demin O.V., Moehren G., Hoek J.B. (1999). Quantification of Short Term Signaling by the Epidermal Growth Factor Receptor. J. Biol. Chem..

[64] Bruggeman F.J., Westerhoff H.V., Hoek J.B., Kholodenko B.N. (2002). Modular Response Analysis of Cellular Regulatory Networks. J. Theor. Biol..

[65] Smirnov P., Kofia V., Maru A., Freeman M., Ho C., El-Hachem N., Adam G.A., Ba-Alawi W., Safikhani Z., Haibe-Kains B. (2018). PharmacoDB: An Integrative Database for Mining in Vitro Anticancer Drug Screening Studies. Nucleic Acids Res..

[66] Rukhlenko O.S., Khorsand F., Krstic A., Rozanc J., Alexopoulos L.G., Rauch N., Erickson K.E., Hlavacek W.S., Posner R.G., Gómez-Coca S. (2018). Dissecting RAF Inhibitor Resistance by Structure-Based Modeling Reveals Ways to Overcome Oncogenic RAS Signaling. Cell Syst..

[67] Kholodenko B.N., Sontag E.D. (2002). Determination of Functional Network Structure from Local Parameter Dependence Data. arXiv.

[68] Bolado-Carrancio A., Rukhlenko O.S., Nikonova E., Tsyganov M.A., Wheeler A., Garcia-Munoz A., Kolch W., von Kriegsheim A., Kholodenko B.N. (2020). Periodic Propagating Waves Coordinate Rhogtpase Network Dynamics at the Leading and Trailing Edges during Cell Migration. Elife.

[69] Aksamitiene E., Kiyatkin A., Kholodenko B.N. (2012). Cross-Talk between Mitogenic Ras/MAPK and Survival PI3K/Akt Pathways: A Fine Balance. Biochem. Soc. Trans..

[70] Molinelli E.J., Korkut A., Wang W., Miller M.L., Gauthier N.P., Jing X., Kaushik P., He Q., Mills G., Solit D.B. (2013). Perturbation Biology: Inferring Signaling Networks in Cellular Systems. PLoS Comput. Biol..

[71] Meunier P.Y., Raynaud C., Guimaraes E., Gueyffier F., Letrilliart L. (2023). Barriers and Facilitators to the Use of Clinical Decision Support Systems in Primary Care: A Mixed-Methods Systematic Review. Ann. Fam. Med..

[72] Kholodenko B.N., Cascante M., Hoek J.B., Westerhoff H.V., Schwaber J. (1998). Metabolic Design: How to Engineer a Living Cell to Desired Metabolite Concentrations and Fluxes. Biotechnol. Bioeng..

[73] Takahashi K., Yamanaka S. (2006). Induction of Pluripotent Stem Cells from Mouse Embryonic and Adult Fibroblast Cultures by Defined Factors. Cell.

[74] Jia D., Jolly M.K., Kulkarni P., Levine H. (2017). Phenotypic Plasticity and Cell Fate Decisions in Cancer: Insights from Dynamical Systems Theory. Cancers.

[75] Moris N., Pina C., Arias A.M. (2016). Transition States and Cell Fate Decisions in Epigenetic Landscapes. Nat. Rev. Genet..

[76] Hafner A., Bulyk M.L., Jambhekar A., Lahav G. (2019). The Multiple Mechanisms That Regulate P53 Activity and Cell Fate. Nat. Rev. Mol. Cell Biol..

[77] Patel R., Islam S.A., Bommareddy R.R., Smalley T., Acevedo-Duncan M. (2020). Simultaneous Inhibition of Atypical Protein Kinase-C and MTOR Impedes Bladder Cancer Cell Progression. Int. J. Oncol..

