# Supplementary material for: Cell State Transition Models Stratify Breast Cancer Cell Phenotypes and Reveal New Therapeutic Targets
Source: Cancers (Basel). 2024 Jun 27;16(13):2354. doi: 10.3390/cancers16132354 (PMC11240448; doi:10.3390/cancers16132354)
Supplement: Supplementary file 1 [file cancers-16-02354-s001.zip › Supplementary Figures.pptx]

## Slide 1
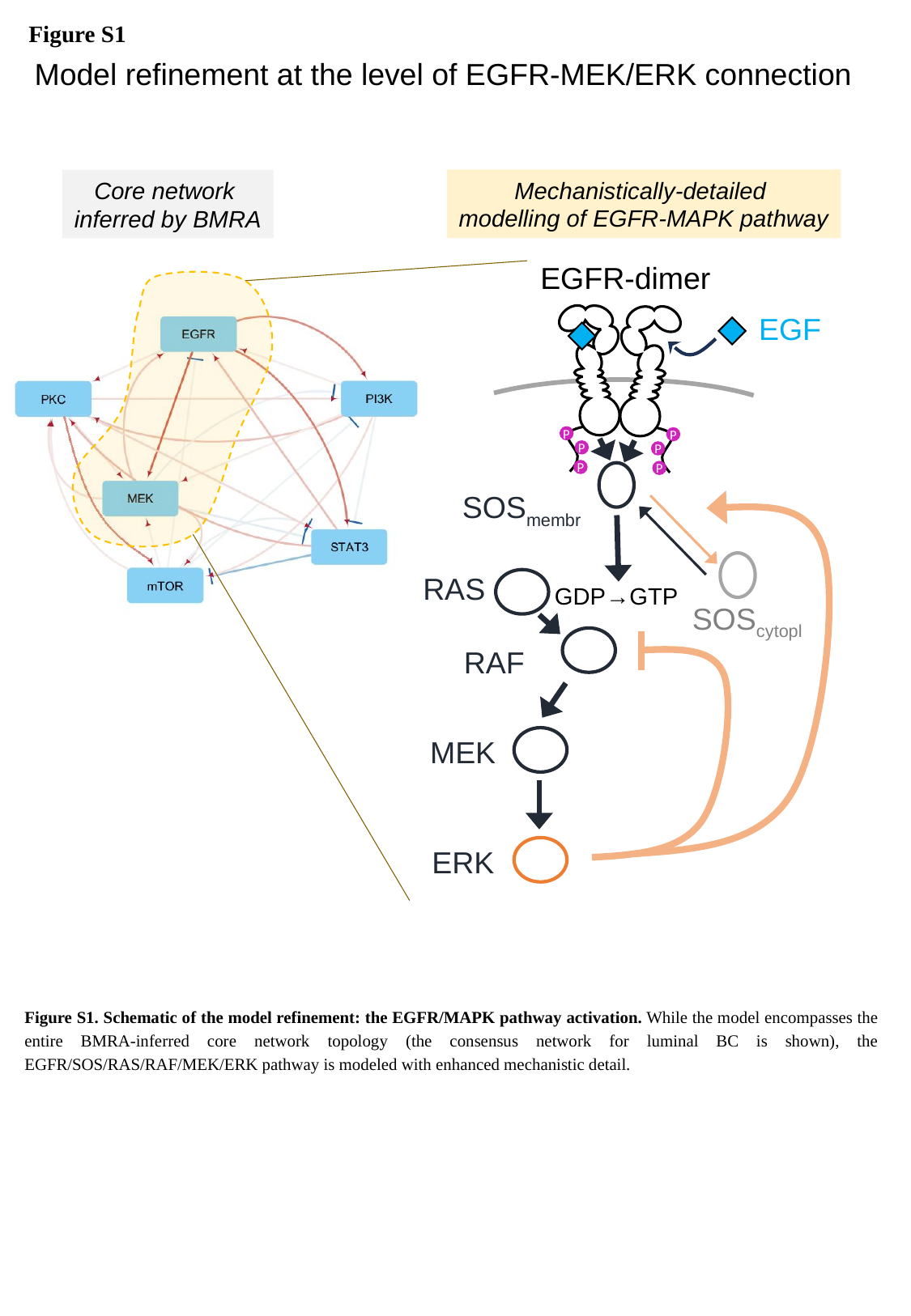

Figure S1
Model refinement at the level of EGFR-MEK/ERK connection
Mechanistically-detailed
modelling of EGFR-MAPK pathway
Core network
inferred by BMRA
EGFR-dimer
EGF
P
P
P
P
P
P
SOSmembr
RAS
GDP→GTP
SOScytopl
RAF
MEK
ERK
Figure S1. Schematic of the model refinement: the EGFR/MAPK pathway activation. While the model encompasses the entire BMRA-inferred core network topology (the consensus network for luminal BC is shown), the EGFR/SOS/RAS/RAF/MEK/ERK pathway is modeled with enhanced mechanistic detail.

## Slide 2
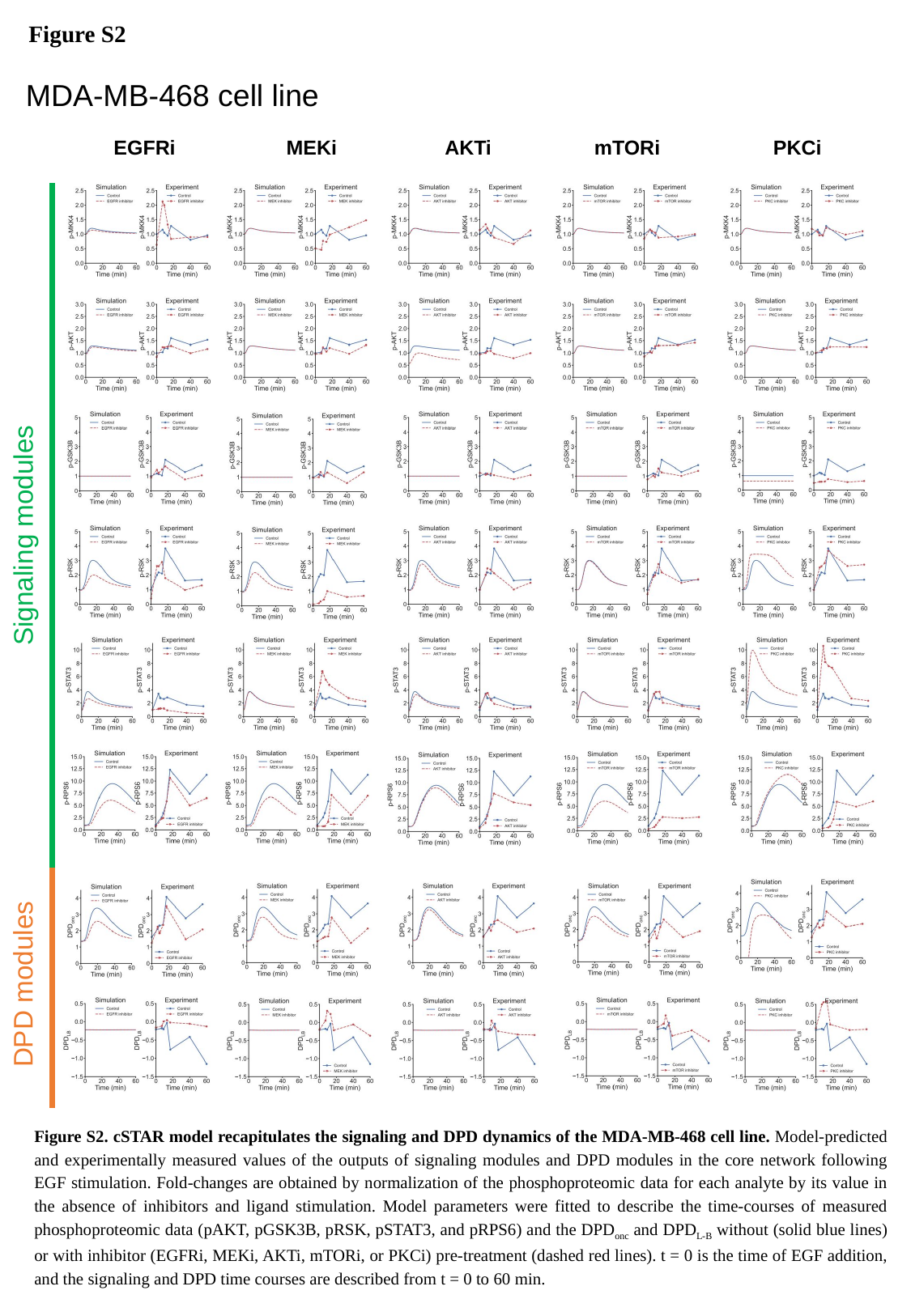

Figure S2
MDA-MB-468 cell line
AKTi
mTORi
PKCi
MEKi
EGFRi
Signaling modules
DPD modules
Figure S2. cSTAR model recapitulates the signaling and DPD dynamics of the MDA-MB-468 cell line. Model-predicted and experimentally measured values of the outputs of signaling modules and DPD modules in the core network following EGF stimulation. Fold-changes are obtained by normalization of the phosphoproteomic data for each analyte by its value in the absence of inhibitors and ligand stimulation. Model parameters were fitted to describe the time-courses of measured phosphoproteomic data (pAKT, pGSK3B, pRSK, pSTAT3, and pRPS6) and the DPDonc and DPDL-B without (solid blue lines) or with inhibitor (EGFRi, MEKi, AKTi, mTORi, or PKCi) pre-treatment (dashed red lines). t = 0 is the time of EGF addition, and the signaling and DPD time courses are described from t = 0 to 60 min.

## Slide 3
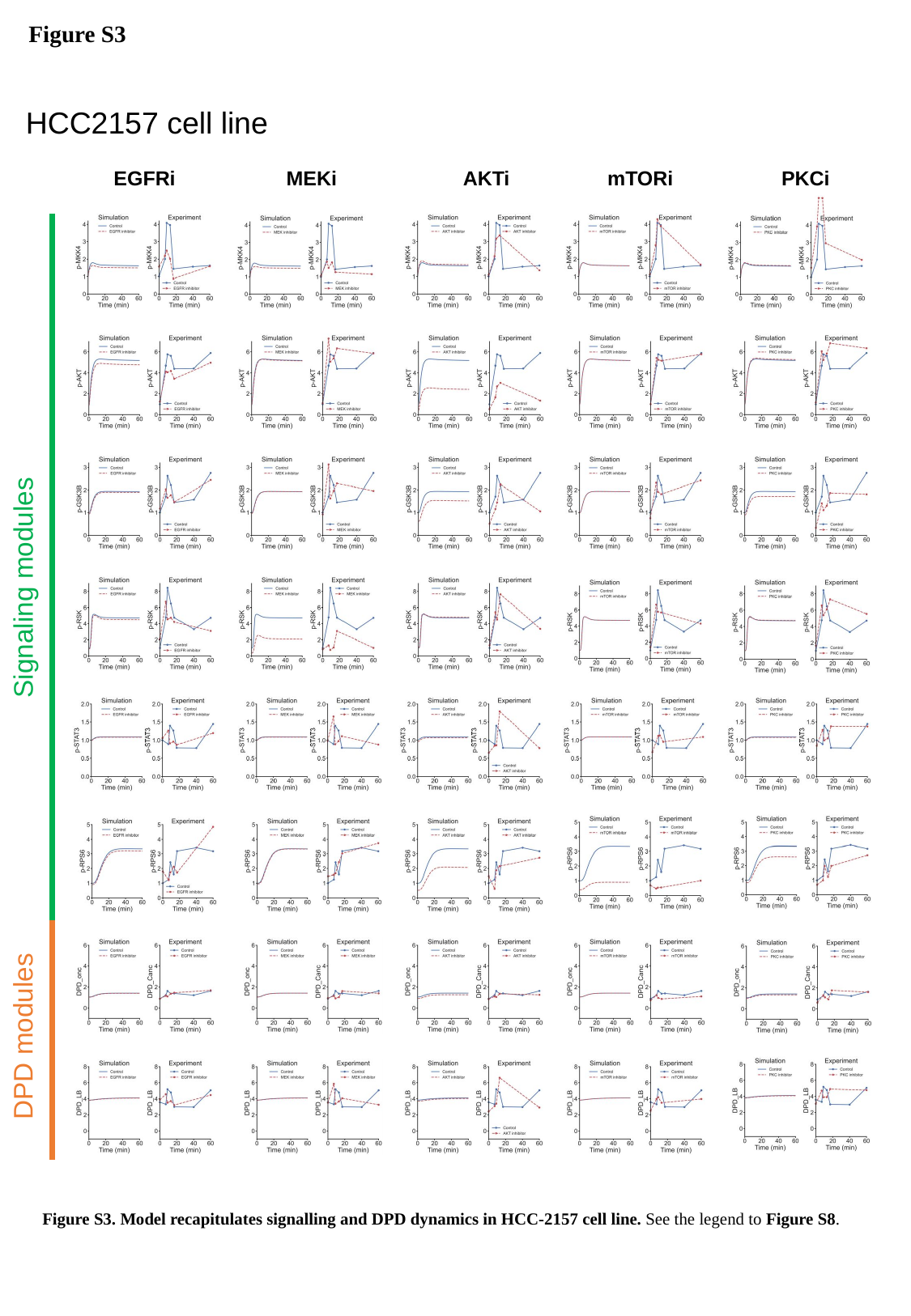

Figure S3
HCC2157 cell line
AKTi
mTORi
PKCi
MEKi
EGFRi
Signaling modules
DPD modules
Figure S3. Model recapitulates signalling and DPD dynamics in HCC-2157 cell line. See the legend to Figure S8.

## Slide 4
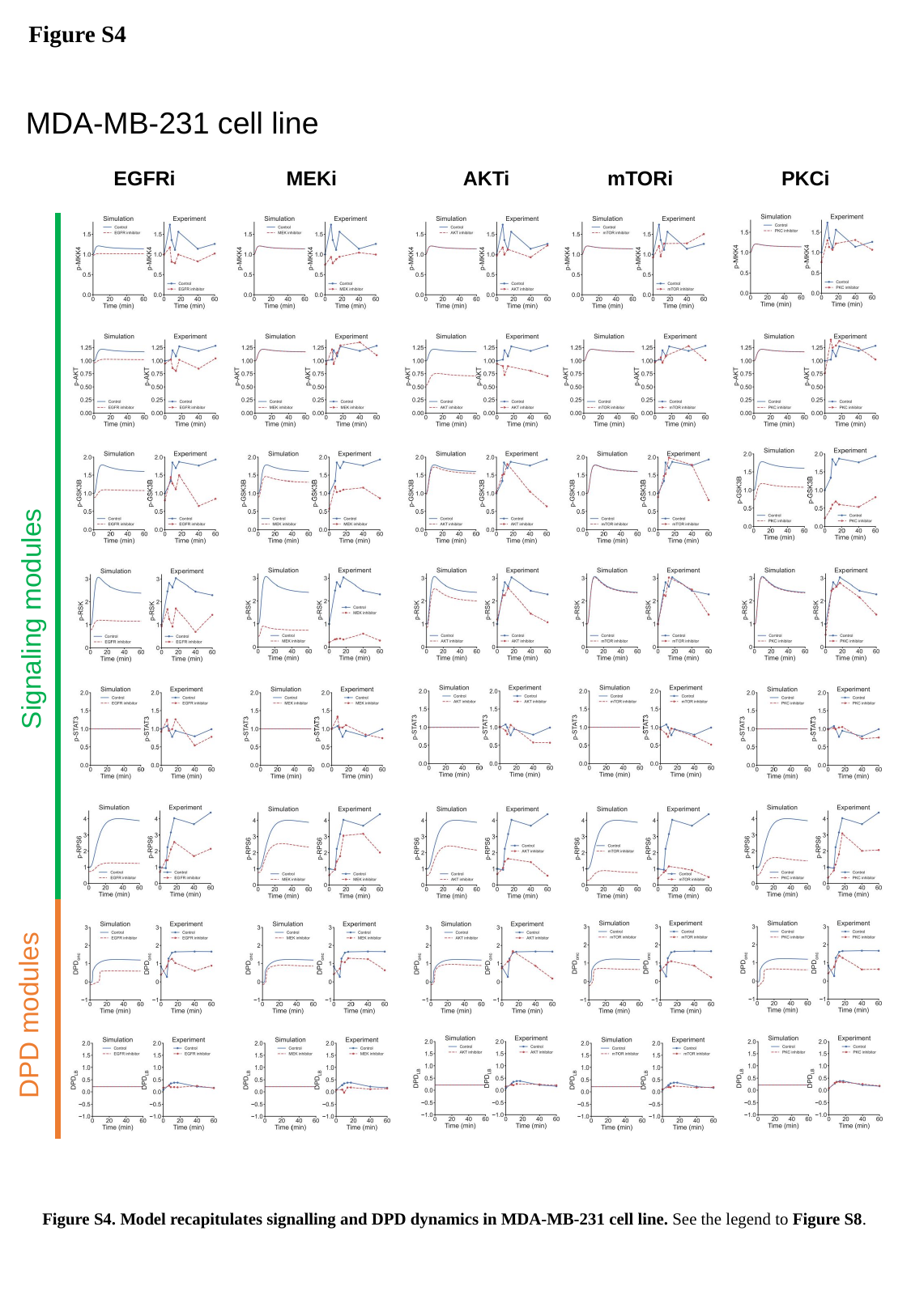

Figure S4
MDA-MB-231 cell line
AKTi
mTORi
PKCi
MEKi
EGFRi
Signaling modules
DPD modules
Figure S4. Model recapitulates signalling and DPD dynamics in MDA-MB-231 cell line. See the legend to Figure S8.

## Slide 5
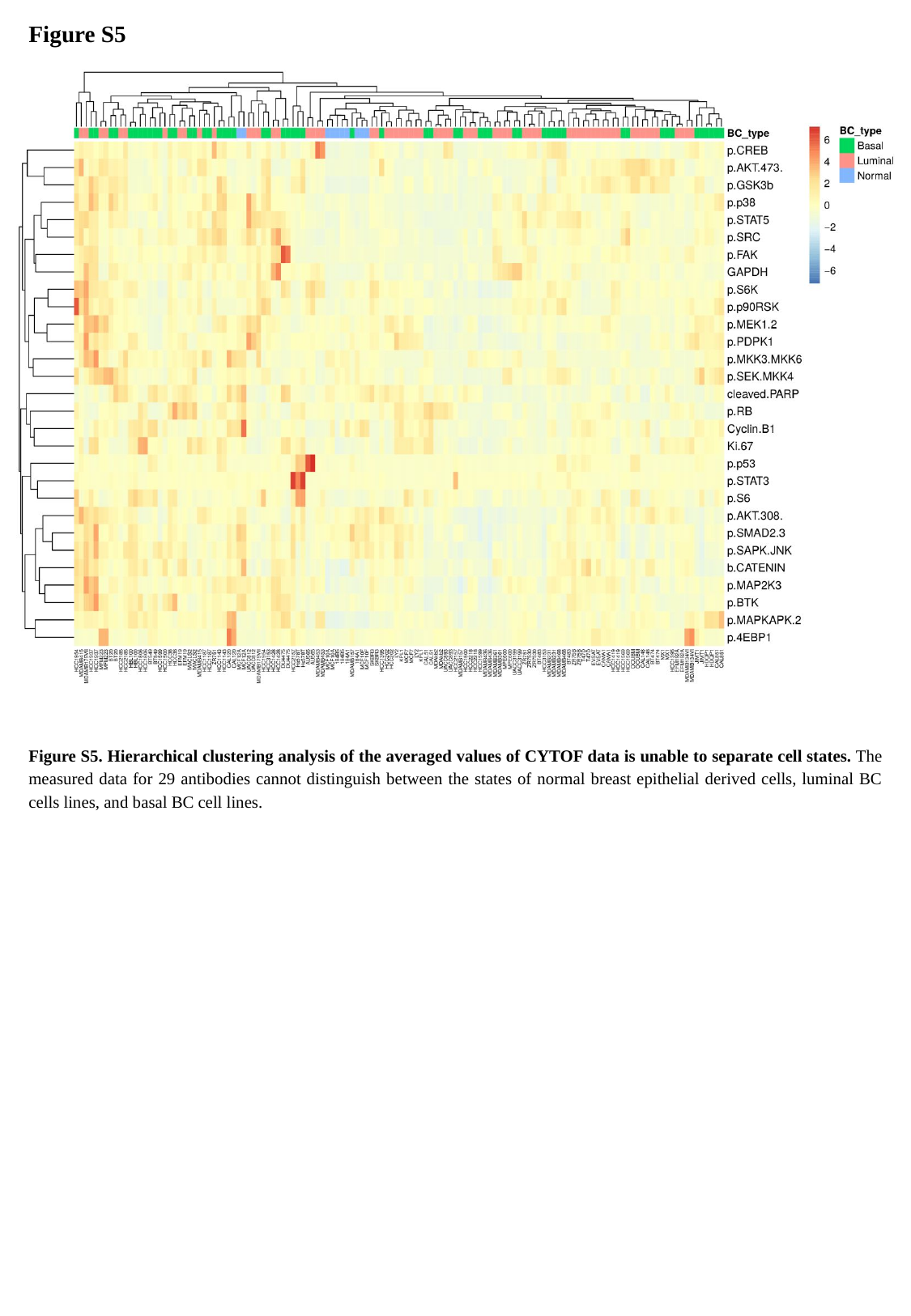

Figure S5
Figure S5. Hierarchical clustering analysis of the averaged values of CYTOF data is unable to separate cell states. The measured data for 29 antibodies cannot distinguish between the states of normal breast epithelial derived cells, luminal BC cells lines, and basal BC cell lines.

## Slide 6
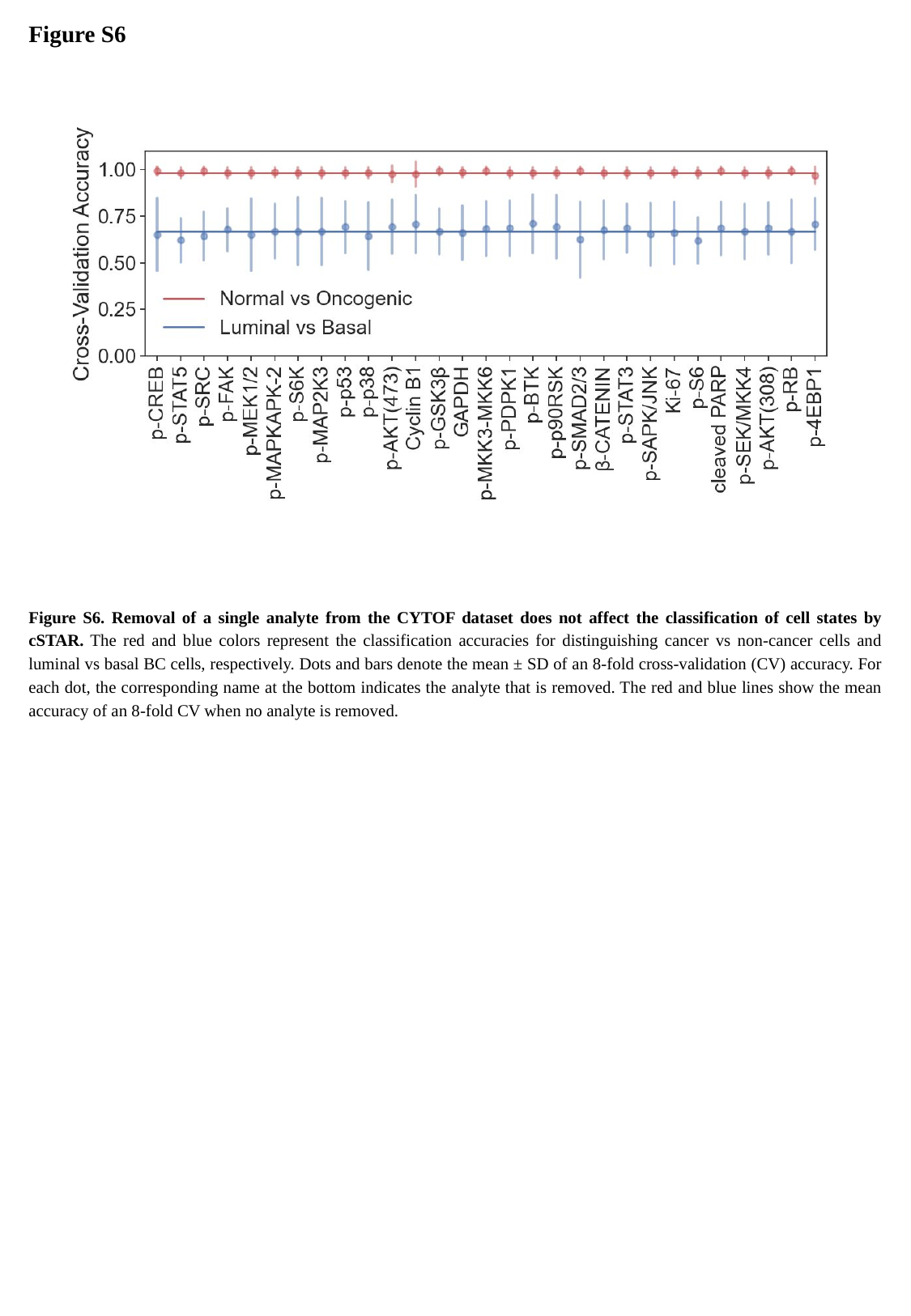

Figure S6
Figure S6. Removal of a single analyte from the CYTOF dataset does not affect the classification of cell states by cSTAR. The red and blue colors represent the classification accuracies for distinguishing cancer vs non-cancer cells and luminal vs basal BC cells, respectively. Dots and bars denote the mean ± SD of an 8-fold cross-validation (CV) accuracy. For each dot, the corresponding name at the bottom indicates the analyte that is removed. The red and blue lines show the mean accuracy of an 8-fold CV when no analyte is removed.

## Slide 7
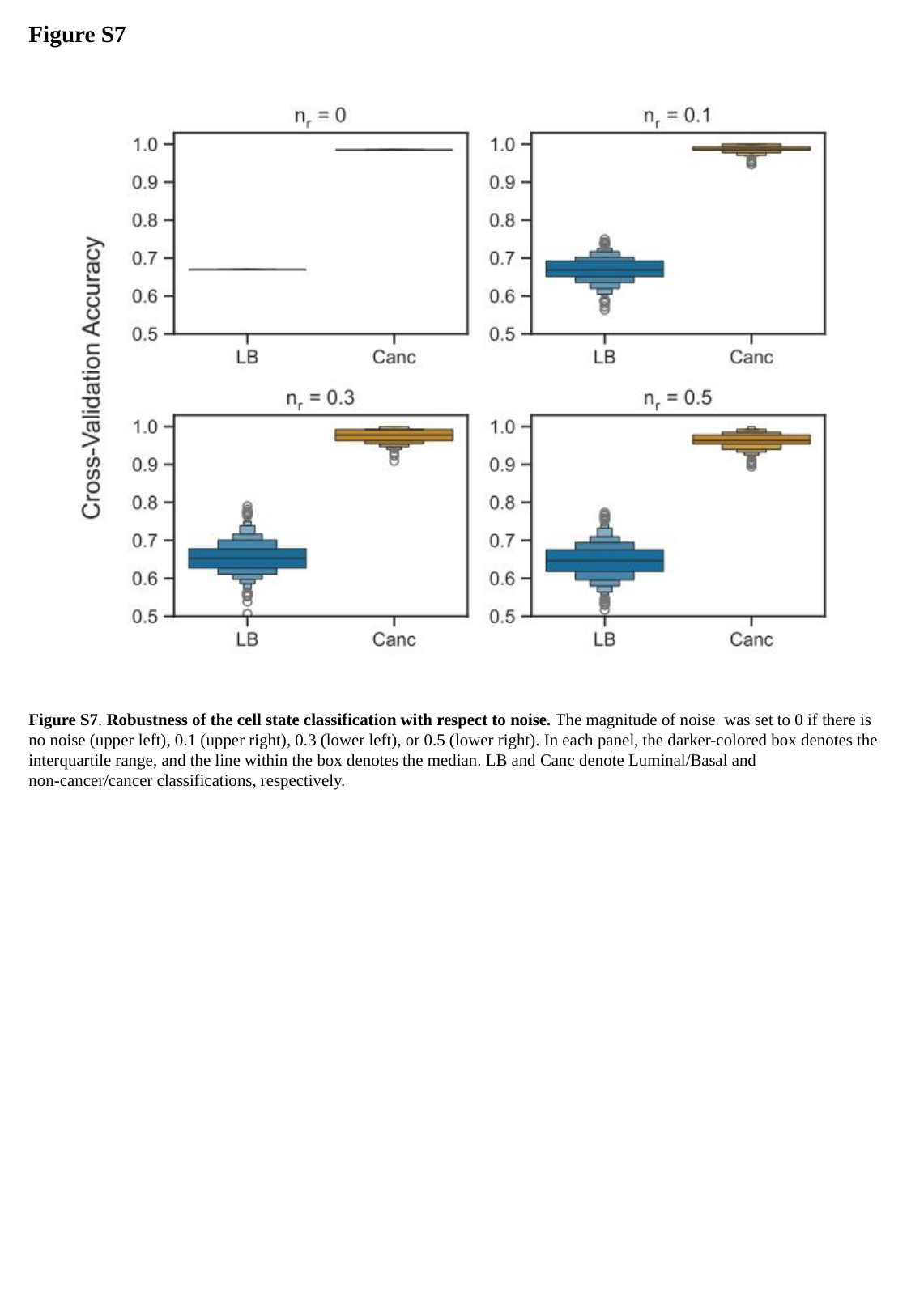

Figure S7

## Slide 8
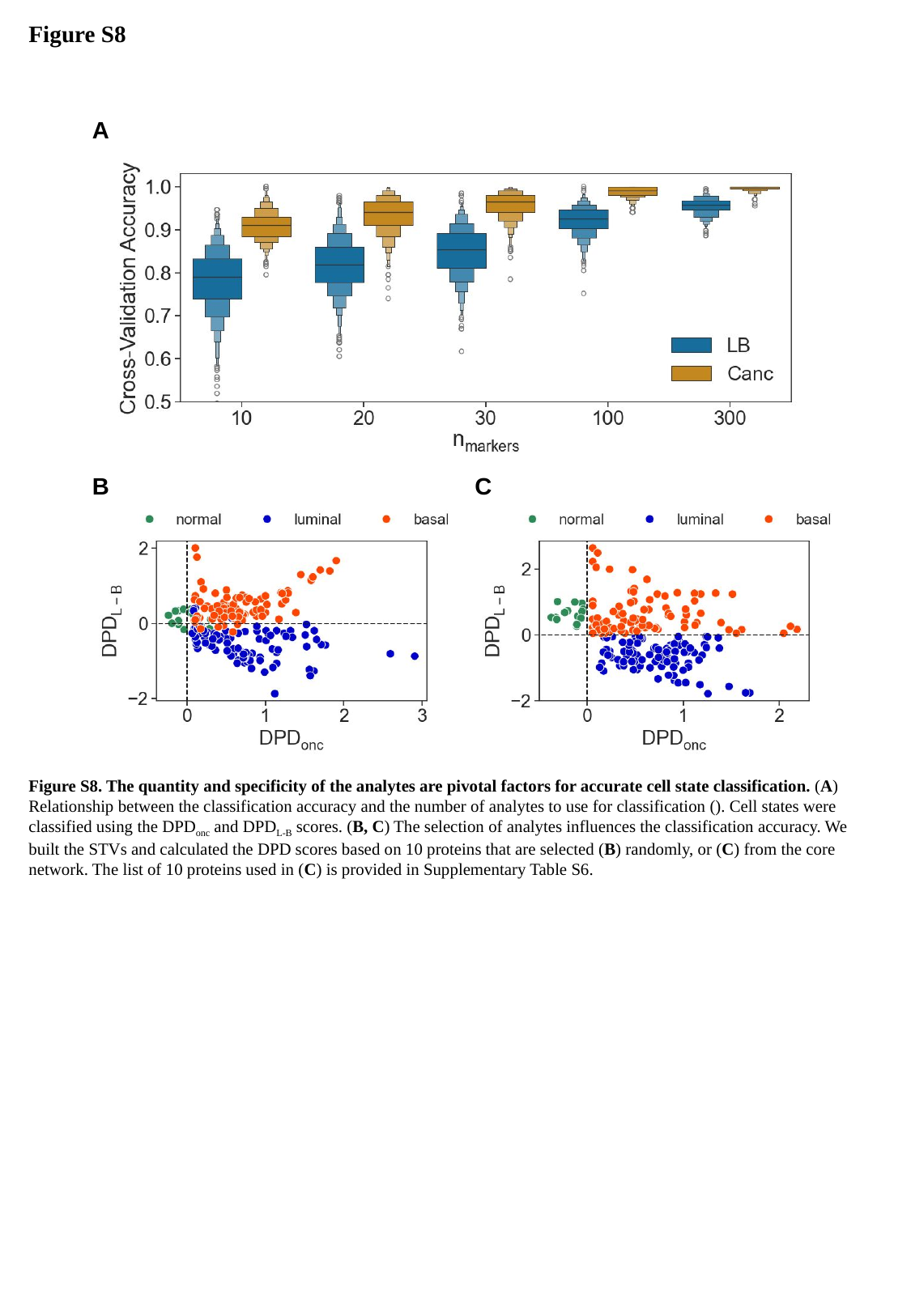

Figure S8
A
C
B

## Slide 9
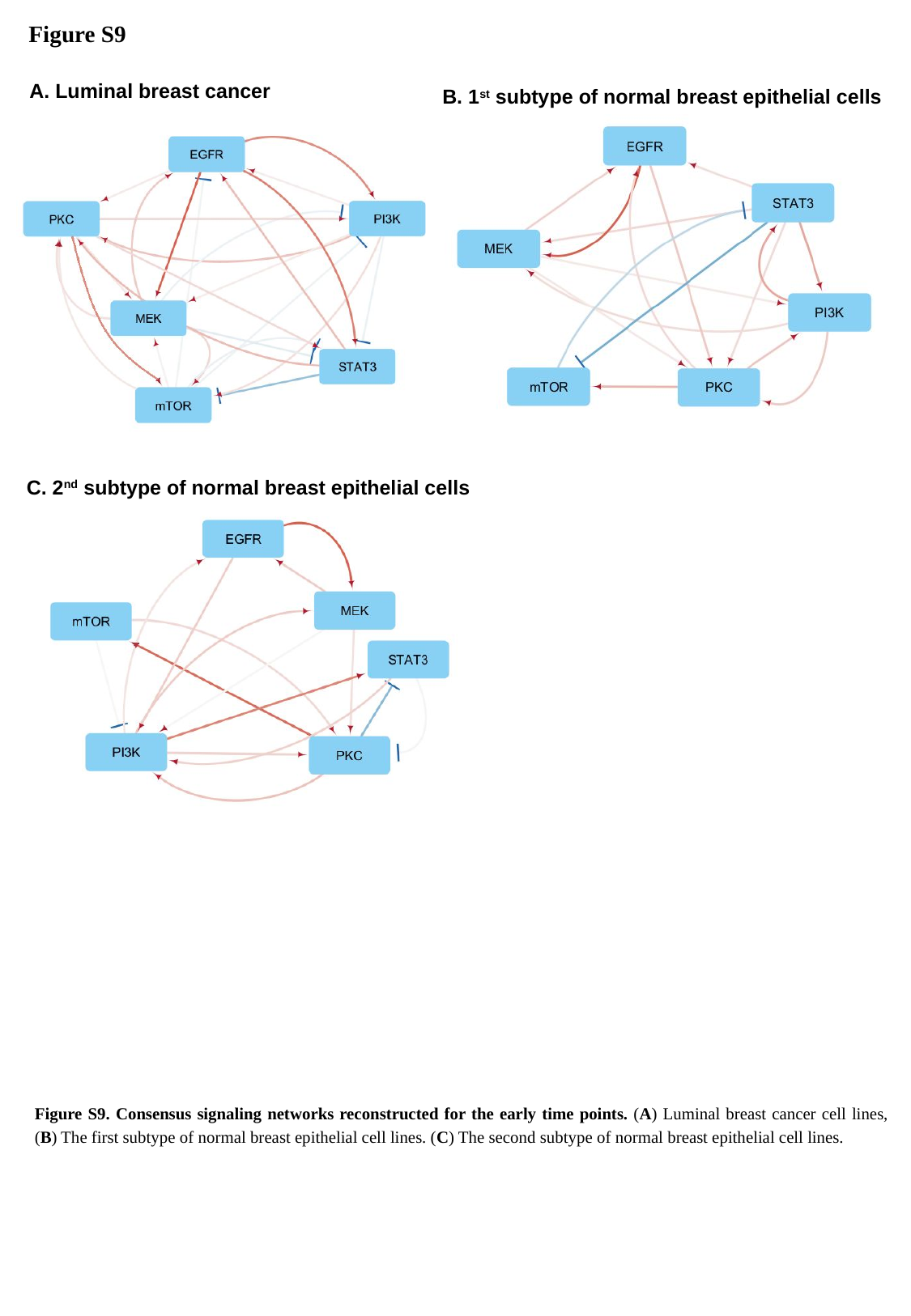

Figure S9
A. Luminal breast cancer
B. 1st subtype of normal breast epithelial cells
C. 2nd subtype of normal breast epithelial cells
Figure S9. Consensus signaling networks reconstructed for the early time points. (A) Luminal breast cancer cell lines, (B) The first subtype of normal breast epithelial cell lines. (C) The second subtype of normal breast epithelial cell lines.

## Slide 10
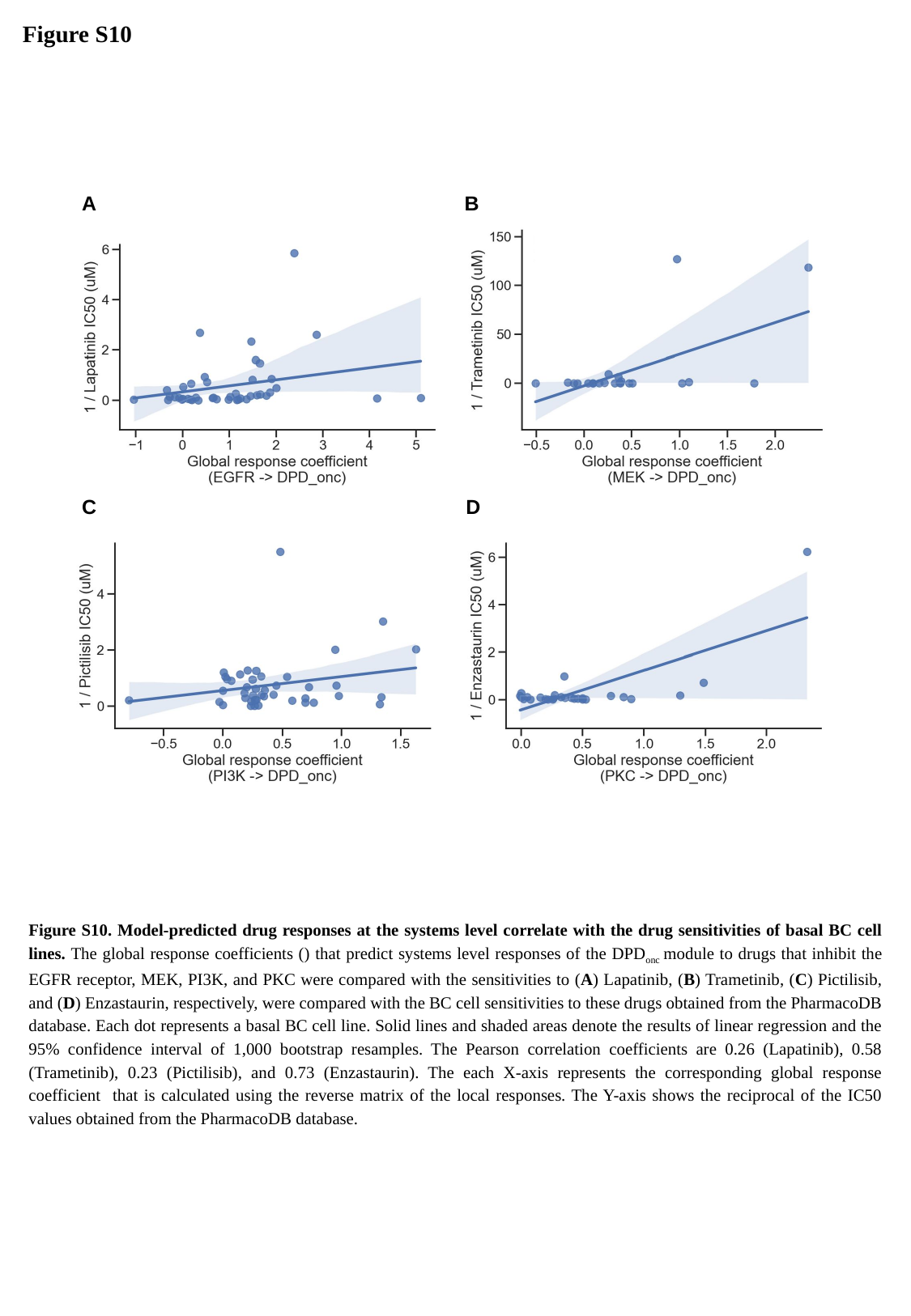

Figure S10
A
B
Lapatinib, 1/IC50 (μM-1)
Trametinib, 1/IC50 (μM-1)
D
C
Enzastaurin, 1/IC50 (μM-1)
Pictilisib, 1/IC50 (μM-1)
