# Supplementary material for: Cell State Transition Models Stratify Breast Cancer Cell Phenotypes and Reveal New Therapeutic Targets
Source: Cancers (Basel). 2024 Jun 27;16(13):2354. doi: 10.3390/cancers16132354 (PMC11240448; doi:10.3390/cancers16132354)
Supplement: Supplementary file 1 [file cancers-16-02354-s001.zip › Table S6.pdf]

Table S6. List of proteins used in Figure S9C.

UniProt Entry	Gene
P00533	EGFR
P31749	AKT1
P31751	AKT2
Q02750	MEK1
P36507	MEK2
P27361	ERK1
P28482	ERK2
P40763	STAT3
P42345	mTOR
P17252	PKC-alpha
